# Inhibition of sulfotransferase SULT2B1 prevents obesity and insulin resistance by regulating energy expenditure and intestinal lipid absorption

**DOI:** 10.1016/j.jbc.2025.110327

**Published:** 2025-05-31

**Authors:** Jingyuan Wang, Gregory Young, Min Zhang, Sonia R. Salvatore, Fu-Ying Qin, Xinran Cai, Meishu Xu, Mengyun Ke, Lingyi Liu, Jong-Won Kim, Pengfei Xu, Bin Yang, Songrong Ren, Ye Feng, Da Yang, Xiaochao Ma, Francisco J. Schopfer, Wen Xie

**Affiliations:** 1 Center for Pharmacogenetics and Department of Pharmaceutical Sciences, University of Pittsburgh, Pittsburgh, Pennsylvania, USA; 2 Department of Pharmacology & Chemical Biology, University of Pittsburgh, Pittsburgh, Pennsylvania, USA; 3 Department of Endocrinology and Metabolic Disease, The First Affiliated Hospital, Zhejiang University School of Medicine, Hangzhou, China

**Keywords:** obesity and type 2 diabetes, insulin resistance, energy expenditure, hepatic steatosis, adipose tissue inflammation

## Abstract

Obesity is a major risk factor for multiple metabolic diseases, including type 2 diabetes mellitus (T2DM) and metabolic dysfunction-associated steatotic liver disease (MASLD). The cholesterol sulfotransferase SULT2B1 is best known for its function in converting cholesterol to cholesterol sulfate. Here, by using the high-fat diet (HFD)-induced obesity model and the genetic obese ob/ob mice, we showed that genetic ablation of Sult2b1 protected mice from developing obesity and related insulin resistance, hepatic steatosis, and adipose tissue inflammation. Loss of Sult2b1 increased energy expenditure without affecting food intake or locomotive activity. The cold exposure test revealed that loss of Sult2b1 promoted thermogenesis in brown adipose tissue, which may have contributed to increased energy expenditure. *In vivo* reconstitution experiments suggested that the loss of Sult2b1 in extrahepatic tissues might have been responsible for the metabolic benefit. Mechanistically, our *in vivo* lipid uptake and metabolomic analyses showed that the Sult2b1KO mice exhibited suppression of intestinal dietary lipid absorption and the consequent downregulation of both systemic fatty acid levels and fatty acid metabolism. Our results suggest that targeting SULT2B1 may represent a novel strategy to combat obesity and related metabolic syndrome.

Obesity is a chronic complex disease defined by an excess accumulation of adipose tissue, imposing deleterious health effects and diagnosed by the body mass index (BMI) (https://www.who.int/news-room/fact-sheets/detail/obesity-and-overweight) (1). According to the latest World Health Organization report, obesity represents a major health concern affecting an estimated 890 million people worldwide and this number has more than doubled in the last 30 years (https://www.who.int/news-room/fact-sheets/detail/obesity-and-overweight). Obesity not only has a major impact on the life quality of patients (2), but also causes considerable economic burden (3). There is a broad range of comorbidities associated with obesity, including the development of insulin resistance leading to type 2 diabetes mellitus (T2DM), metabolic dysfunction-associated steatotic liver disease (MASLD), cardiovascular diseases, and an extensive list of cancers (4, 5, 6). An increasing number of studies have investigated various genes and signaling pathways that regulate adipogenesis and lipolysis, insulin resistance, thermogenesis, dietary lipid absorption, appetite, and adipose tissue inflammation (7). Continued understanding of the pathogenesis of obesity and insulin resistance will help to develop more effective therapies.

SULT2B1a and SULT2B1b are encoded by the sulfotransferase (SULT) 2B1 gene, with SULT2B1b being expressed about 7-fold times higher and more ubiquitously than SULT2B1a (8). As a member of the sulfotransferase family, SUL2B1 sulfonates 3β-hydroxysteroids such as cholesterol, oxysterols, dehydroepiandrosterone (DHEA), and pregnenolone (8, 9, 10, 11). SULT2B1b gain of function has been reported to inhibit hepatic lipogenesis and gluconeogenesis. SULT2B1b inhibits hepatic lipogenesis by sulfonating 25-hydroxycholesterol (25HC), an endogenous ligand for the lipogenic nuclear receptor Liver X Receptor (LXR) (12), to form 25-hydroxycholesterol-3-sulfate (25HC3S) (13, 14). We reported that SULT2B1b or its enzymatic product cholesterol sulfate inhibited hepatic gluconeogenesis by targeting the hepatocyte nuclear factor 4α (HNF4α) and alleviated metabolic abnormalities in obese mice (15). Mechanistically, cholesterol sulfate and SULT2B1b inhibited gluconeogenesis by suppressing the expression of acetyl coenzyme A (acetyl-CoA) synthetase, leading to decreased acetylation and nuclear exclusion of HNF4α (15). Our subsequent study showed that the *SULT2B1b* gene is a transcriptional target of HNF4α, establishing SULT2B1b as a part of the negative feedback to limit hepatic gluconeogenesis since the enzymatic action of SULT2B1b leads to the downregulation of HNF4α-mediated gluconeogenesis (16).

In this study, we discovered unexpectedly that loss of Sult2b1 also protected mice from developing obesity, as well as the related liver steatosis, insulin resistance, and adipose tissue inflammation, which may have been accounted for by the increase in energy expenditure and inhibition of intestinal lipid uptake. Our results suggest that inhibiting SULT2B1 may be a promising approach to combat obesity and its comorbidities.

## Results

### Sult2b1 ablation protects mice from developing obesity

**Figure 1:**
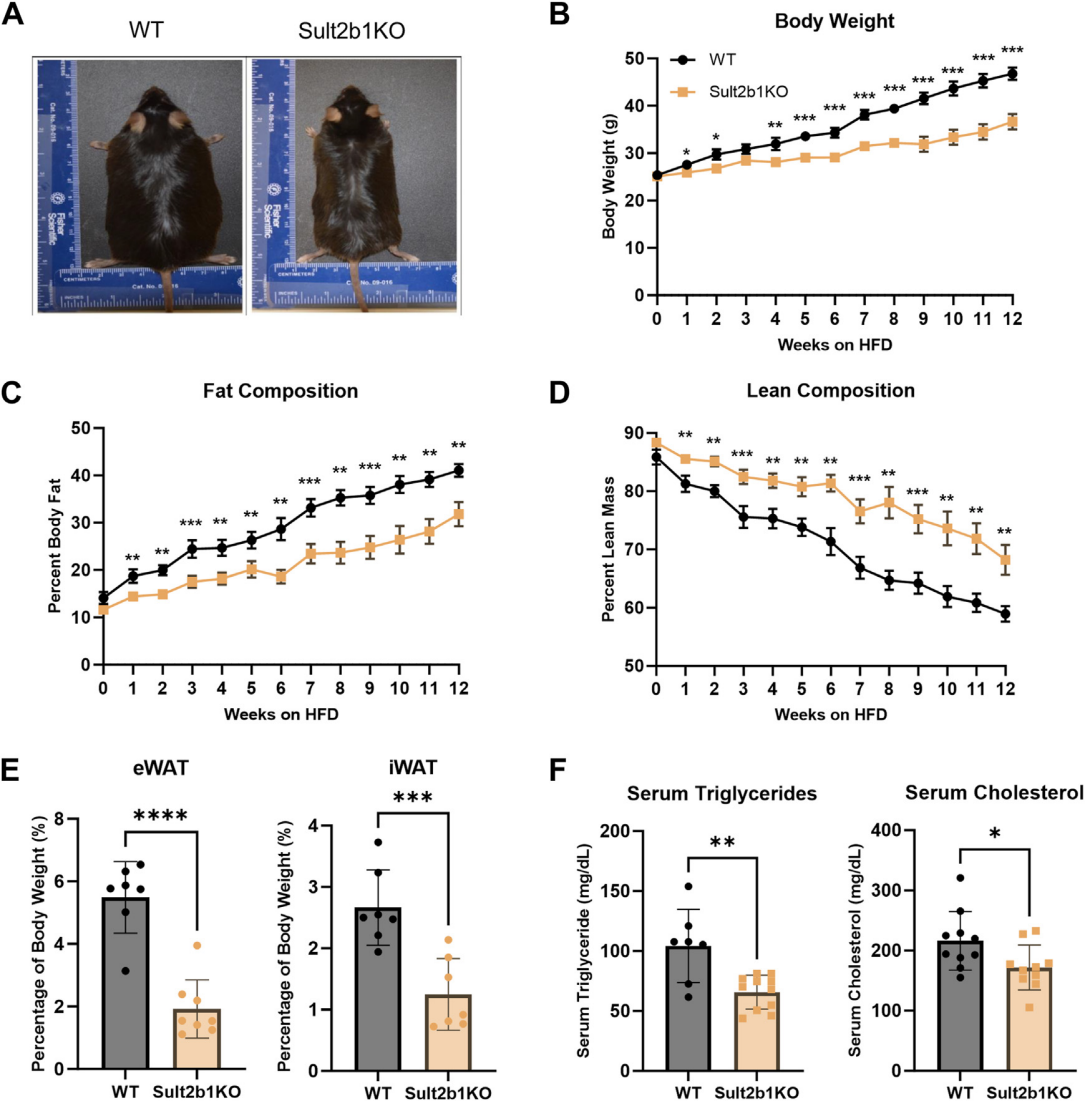
**Sult2b1 ablation protects mice from developing obesity**. Eight-week-old male WT and Sult2b1KO mice were fed with HFD for 12 weeks n = 7 to 8. *A*, representative photograph of mice at the completion of HFD feeding. *B*, time course of body weight while mice were under HFD feeding. *C-D*, body fat mass composition (*C*) and lean mass composition (*D*) measured by Echo-MRI. *E*, weights of eWAT and iWAT as percentages of body weight. *F*, serum triglyceride and cholesterol levels at the completion of HFD feeding. Data is represented as mean ± SD. Significance analysis was performed with unpaired Student’s *t* test. ∗*p* < 0.05; ∗∗*p* < 0.01; ∗∗∗*p* < 0.001; ∗∗∗∗*p* < 0.0001.

We have previously reported that hepatic overexpression of Sult2b1 in transgenic mice or treatment with Sult2b1-derived metabolite cholesterol sulfate protected mice from obesity and associated metabolic syndrome (15). To our surprise, we found the loss-of-function Sult2b1 knockout (Sult2b1KO) mice were also protected from obesity. When fed the control diet, Sult2b1KO showed a modest but significantly lower body weight after 10 weeks of age, while the liver to body weight ratio and adipose tissue to body weight ratio were not different from the WT mice (Fig. S1). In the diet-induced obesity (DIO) model, WT and Sult2b1KO mice were fed with a HFD for 12 weeks and monitored for their body weight and body composition. After 12 weeks of HFD-feeding, WT mice developed obvious obesity while Sult2b1KO mice were visibly smaller (Fig. 1*A*). Sult2b1KO mice displayed significantly less weight gain than their WT counterparts, who had similar body weights at the beginning of HFD feeding (Fig. 1*B*). Body composition analysis showed that as the HFD feeding progressed, the Sult2b1KO mice gained significantly less fat (Fig. 1*C*), with a concomitant increase in lean mass composition (Fig. 1*D*). Necropsy at the end of the experiment showed that the relative epididymal white adipose tissue (eWAT) and inguinal white adipose tissue (iWAT) weights of the Sult2b1KO mice were significantly less than WT mice (Fig. 1*E*). Serum biochemistry showed decreased circulating triglyceride and cholesterol levels in the Sult2b1KO mice, indicating ameliorated hyperlipidemia (Fig. 1*F*).

In an independent model of obesity, we bred the Sult2b1KO allele into the genetically obese ob/ob mice to create ob/ob mice deficient in Sult2b1, termed obsk mice. A similar pattern of attenuation of obesity and improved body composition was observed in obsk mice compared to the ob/ob mice, including smaller body size (Fig. S2*A*), decreased body weight (Fig. S2*B*), decreased fat mass and increased lean mass (Fig. S2*C*), decreased eWAT without affecting iWAT (Fig. S2*D*), and decreased serum levels of triglycerides and cholesterol (Fig. S2*E*). These results demonstrated that loss of Sult2b1 protects mice from developing obesity and hyperlipidemia.

### Sult2b1 null mice are protected from insulin resistance

**Figure 2:**
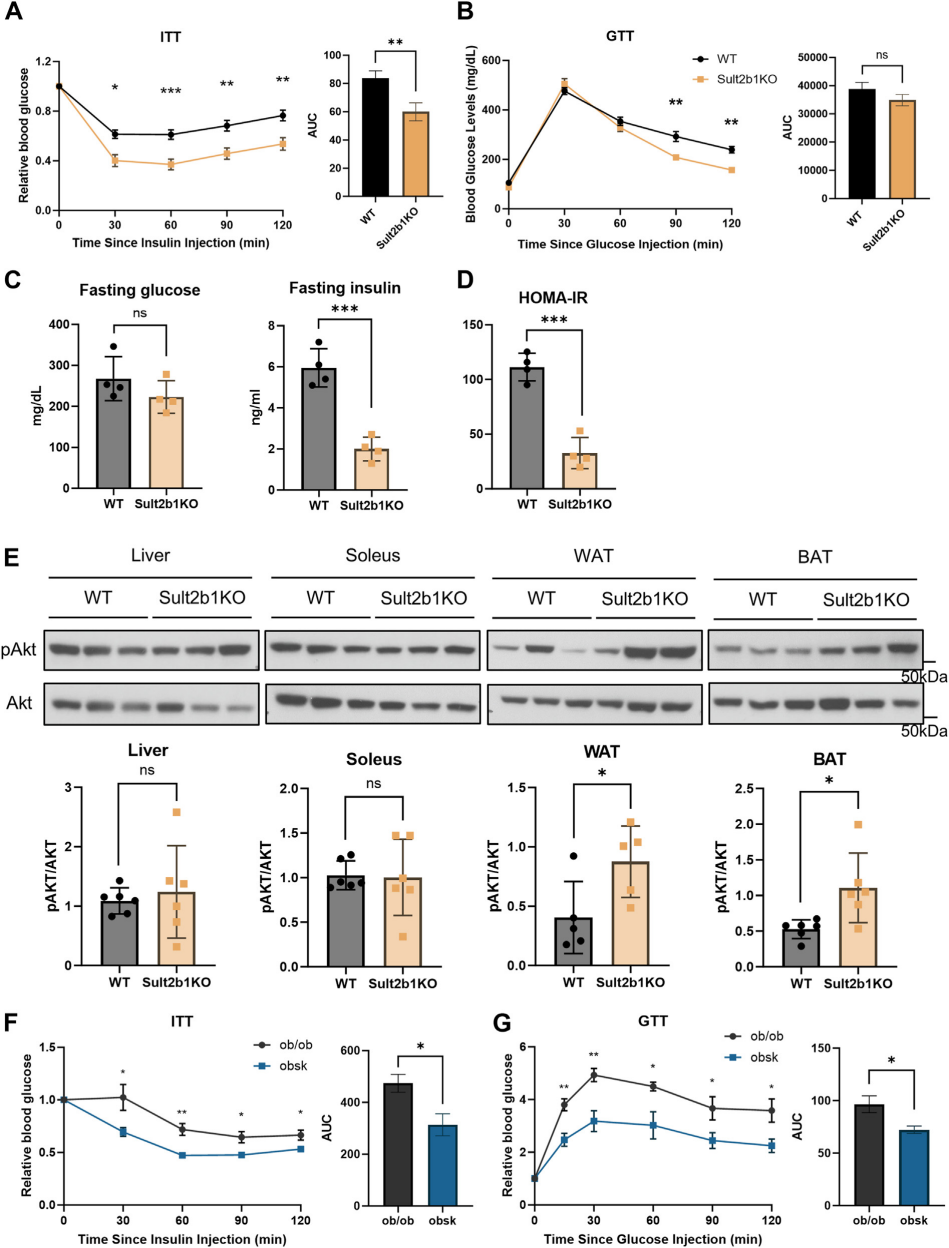
**Sult2b1 null mice are protected from insulin resistance.***A-B*, blood glucose levels during ITT (*A*) and GTT (*B*) of WT and Sult2b1KO mice after 8 weeks of HFD feeding. Bar graphs show the areas under curve (AUC). n = 10 to 12. *C-D*, fasting glucose and insulin levels (*C*) and HOMA-IR level (*D*) of WT and Sult2b1KO mice after 12 weeks of HFD feeding. n = 4. *E*, mice that have been fed with HFD for 12 weeks were intraperitoneally injected with 0.75U/kg insulin and Akt phosphorylation in the liver, skeletal muscle soleus, eWAT, and BAT was evaluated by Western blotting. Quantifications of two independent cohorts are shown by bar graphs below. n = 6. *F-G*, blood glucose levels during ITT (*F*) and GTT (*G*) of 13-week-old ob/ob and obsk mice. Bar graphs show the AUC of the ITT and GTT tests. n = 7 to 8. Data is represented as mean ± SD. Significance analysis was performed with unpaired Student’s *t* test. ∗*p* < 0.05; ∗∗*p* < 0.01; ∗∗∗*p* < 0.001. ns, no significance.

Insulin resistance is frequently accompanied with a metabolic disorder in obese mice and patients. We next examined whether the HFD-fed Sult2b1KO mice were protected from insulin resistance. When subjected to the insulin tolerance test (ITT) and glucose tolerance test (GTT), Sult2b1KO mice fed with HFD for 12 weeks exhibited a better sensitivity to insulin injection (Fig. 2*A*) and improved glucose tolerance (Fig. 2*B*). Meanwhile, Sult2b1KO mice also showed significantly lower fasting blood insulin levels (Fig. 2*C*) and insulin resistance index homeostatic model assessment-insulin resistance (HOMA-IR) (Fig. 2*D*). To gain insight into the improved insulin sensitivity, we evaluated the insulin-induced Akt phosphorylation in four major metabolic tissues: liver, WAT, brown adipose tissue (BAT), and soleus/skeletal muscle. Increased Akt phosphorylation was observed in WAT and BAT, but not in the liver or soleus of Sult2b1KO mice (Fig. 2*E*), suggesting an adipose tissue-specific improvement of insulin sensitivity. The obsk mice also exhibited improved performance in their ITT (Fig. 2*F*) and GTT (Fig. 2*G*) tests. These results demonstrated that loss of Sult2b1 protects mice from developing insulin resistance.

### Sult2b1 ablation protects mice from hepatic steatosis

**Figure 3:**
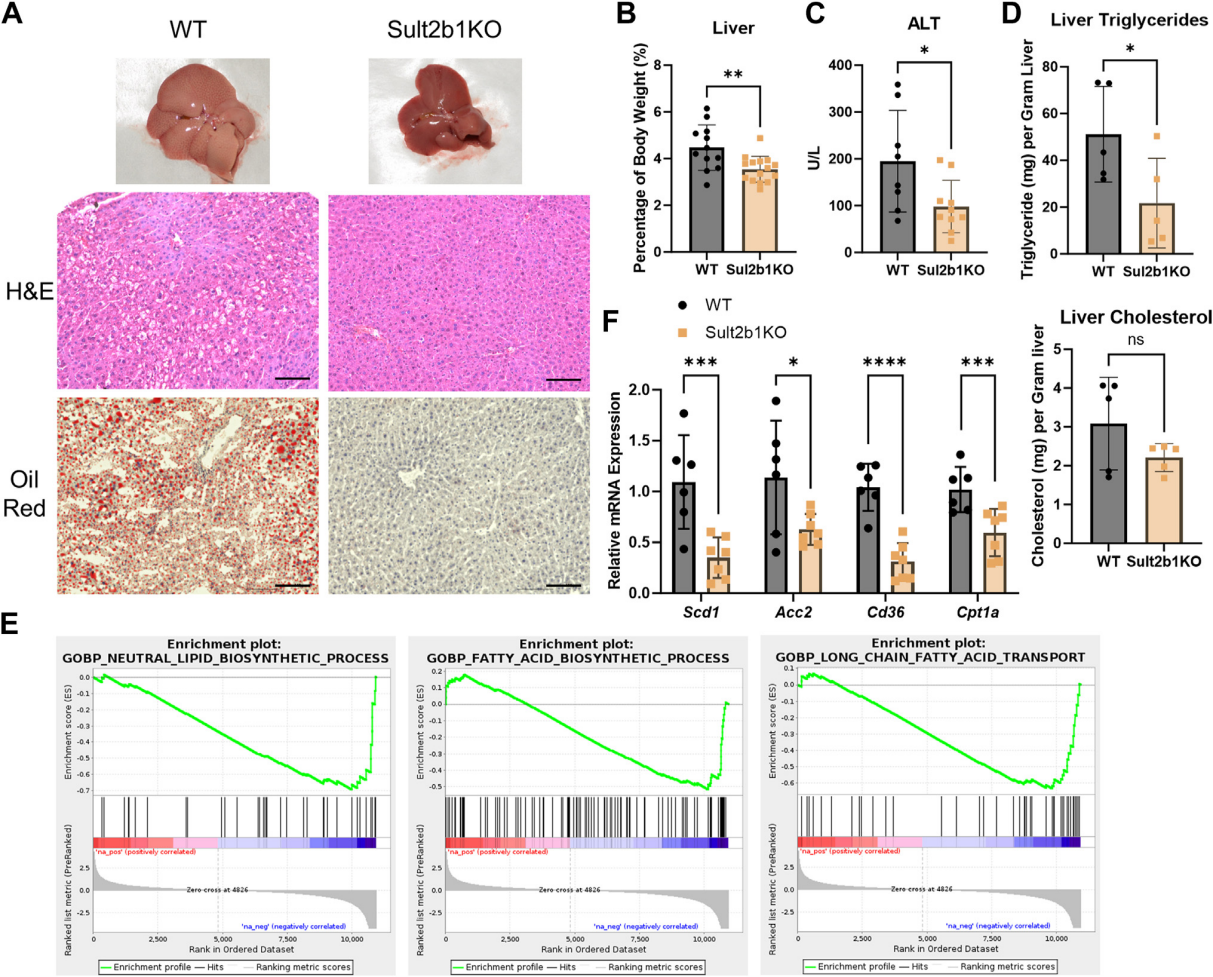
**Sult2b1 ablation protects mice from hepatic steatosis**. WT and Sult2b1KO mice and the HFD treatment were the same as described in Figure 1. *A*, photograph of the liver and H&E staining and Oil Red O staining of the liver sections after 12 weeks of HFD feeding. Scale bar, 100 μm. *B-D*, relative liver weight (*B*), serum ALT level (*C*), and hepatic triglycerides and cholesterol (*D*) levels after 12 weeks of HFD feeding. *E*, GSEA of RNA-seq data derived from HFD-fed mouse livers. Sult2b1KO vs. WT. *F*, relative mRNA expression of lipid metabolism-related genes in the livers. n = 6. Data is represented as mean ± SD. Significance analysis was performed with unpaired Student’s *t* test and multiple *t*-tests. ∗*p* < 0.05; ∗∗*p* < 0.01; ∗∗∗*p* < 0.001.

Another hallmark of obesity and metabolic syndrome is hepatic steatosis. We observed that the HFD-fed Sult2b1KO mice also showed a relief of hepatic steatosis, as evidenced by the less pale liver gross appearance, reduced lipid cavities in histology, and decreased Oil Red O staining (Fig. 3*A*). Consistently, the liver-to-body weight ratio (Fig. 3*B*), serum ALT level (Fig. 3*C*), and liver triglyceride level (Fig. 3*D*) were all decreased in Sult2b1KO mice. The liver cholesterol level showed a trend of decrease, but the change was not statistically significant (Fig. 3*D*). Mechanistically, the gene set enrichment analysis (GSEA) of our RNA-seq results revealed downregulation of hepatic genes involved in lipid biosynthesis and fatty acid transport in HFD-fed Sult2b1KO mice (Fig. 3*E*). The suppressions of genes involved in lipogenesis (*Scd1* and *Acc2*), fatty acid uptake (*Cd36*), and fatty acid oxidation (*Cpt1a*) (Fig. 3*F*) were verified by real-time PCR. Compared to the ob/ob mice, the liver of obsk mice also showed decreased Oil Red O staining (Fig. S3*A*) and decreased liver-to-body weight ratio (Fig. S3*B*) without affecting serum ALT level (Fig. S3*C*), liver lipid contents (Fig. S3*D*), or the expression of *Scd1*, *Cd36*, *Acc2*, or *Cpt1a* (Fig. S3*E*).

### The metabolic benefits of Sult2b1 ablation occur independent of hepatic fatty acid uptake transporter Cd36 suppression and cannot be attributed solely to the loss of Sult2b1 in the liver

Cd36 is well known for its fatty acid uptake function. The downregulation of Cd36 in the liver of HFD-fed Sult2b1KO mice, first suggested by RNA-seq analysis, was further verified by Western blotting (Fig. S4*A*) and immunofluorescence (Fig. S4*B*). To determine whether the suppression of Cd36 played a mechanistical role in the exhibition of metabolic phenotypes, we reconstituted the hepatic expression of Cd36 by crossbreeding the Sult2b1KO mice with our previously reported liver-specific Cd36 transgenic (CD36 Tg) mice (17). The reconstitution of Cd36 in the liver was verified by qPCR (Fig. S4*C*) and IF (Fig. S4*D*). Upon a 16-week HFD feeding, the Sult2b1KO-CD36 Tg mice were indistinguishable from their Sult2b1KO counterparts in their body weight gain (Fig. S4*E*), body compositions (Fig. S4*F*), eWAT and iWAT (Fig. S4*G*), GTT and ITT (Fig. S4*H*), and the liver to body weight ratio (Fig. S4*I*). These results suggested that the downregulation of hepatic CD36 was unlikely responsible for the metabolic benefits observed in the Sult2b1KO mice.

**Figure 4:**
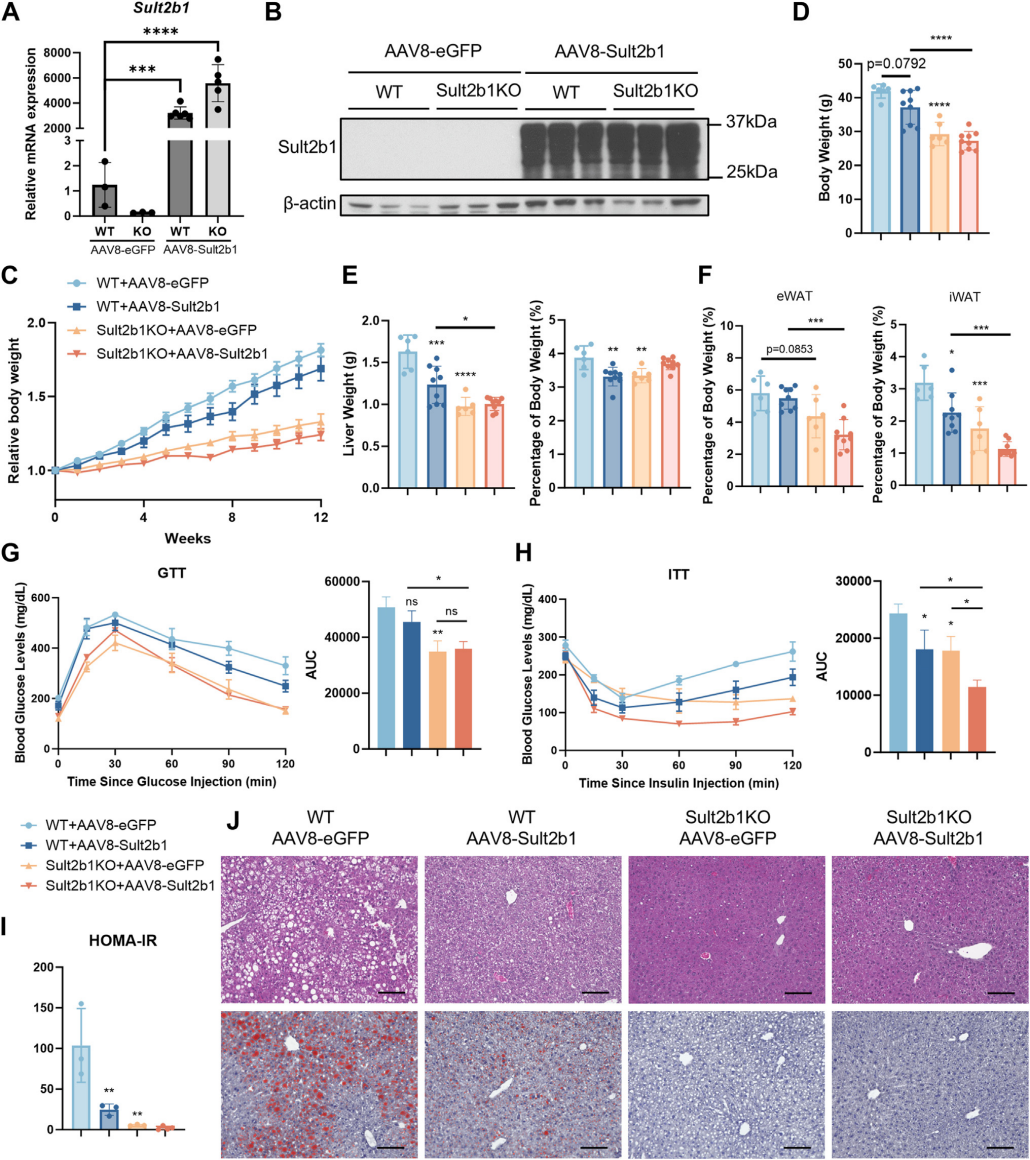
**Hepatic Sult2b1 is not responsible for the metabolic benefit of Sult2b1 ablation**. Eight weeks old male WT and Sult2b1KO mice were infected with the control AAV8-TBG-eGFP or AAV8-TBG-Sult2b1 and fed with HFD for 12 weeks. *A*, relative mRNA expression of Sult2b1 in the liver as measured by real-time PCR. n = 3 to 6. KO, Sult2b1KO. *B*, Sult2b1 protein levels in the liver as measured by Western blotting. β-actin was used as a loading control. n = 3. *C-D*, body weight during HFD feeding (*C*) and at the completion of HFD feeding (*D*). *E*, liver weight and relative liver weight at the completion of HFD feeding. *F*, weights of eWAT and iWAT as the percentages of body weight at the completion of HFD feeding. *G-H*, GTT (*G*) and ITT (*H*) at the completion of HFD feeding. AUC was measured (right panel). *I*, HOMA-IR calculated from fasted glucose and insulin levels. n = 3. *J*, H&E staining and Oil Red O staining of the liver sections at the completion of HFD feeding. Scale bar, 100 μm. Data are represented as mean ± SD. Significance analysis was performed with one-way ANOVA (multiple comparisons). ∗*p* < 0.05; ∗∗*p* < 0.01; ∗∗∗∗*p* < 0.0001. ns, no significance. Without indication, the analysis is comparing to WT + AAV8-eGFP group.

The use of whole-body Sult2b1KO mice prevented us from pinpointing the cellular source of Sult2b1 in the phenotypic exhibition. Knowing the liver is an essential metabolic organ that controls energy metabolism (18), and that the previous metabolic functions of Sult2b1 have been mostly characterized in the liver (15, 16, 19), we then explored whether it is the loss of hepatic Sult2b1 that is responsible for the metabolic benefits. In this experiment, we reconstituted the hepatocyte expression Sult2b1 by infecting the Sult2b1KO mice with an adeno-associated virus 8 expressing Sult2b1 under the control of hepatocyte-specific thyroxine binding globulin (TBG) promoter (AAV8-TBG-Sult2b1) before subjecting the mice to 12-weeks HFD feeding. The overexpression of Sult2b1 in the liver was verified and sustained until the end of HFD feeding as shown by qPCR (Fig. 4*A*) and Western blotting (Fig. 4*B*). Consistent with our previous report (15), Sult2b1 overexpression in the liver protected WT mice from gaining weight during HFD feeding (Fig. 4*C*), which was also evidenced by lower body weight (Fig. 4*D*), liver weight and liver to body weight ratio (Fig. 4*E*), and iWAT weight (Fig. 4*F*). Liver-specific reconstitution of Sult2b1 in Sult2b1KO mice led to further decrease or no change in body and tissue weights, which were still significantly lower than their AAV8-TBG-Sult2b1 infected WT counterparts (Fig. 4, *C–F*). Similarly, liver-specific reconstitution of Sult2b1 failed to abolish improved insulin sensitivity, as shown by GTT (Fig. 4*G*), ITT (Fig. 4*H*). The HOMA-IR index was not different between Sult2b1KO mice with or without the Sult2b1 reconstitution (Fig. 4*I*). The attenuation of liver steatosis in AAV8-TBG-Sult2b1 infected Sult2b1KO mice remained obvious (Fig. 4*J*). Taken together, our results suggested that the loss of Sult2b1 in the hepatocytes was unlikely responsible for the metabolic benefits observed in Sult2b1KO mice.

### Sult2b1 deficiency attenuates adipose inflammation

**Figure 5:**
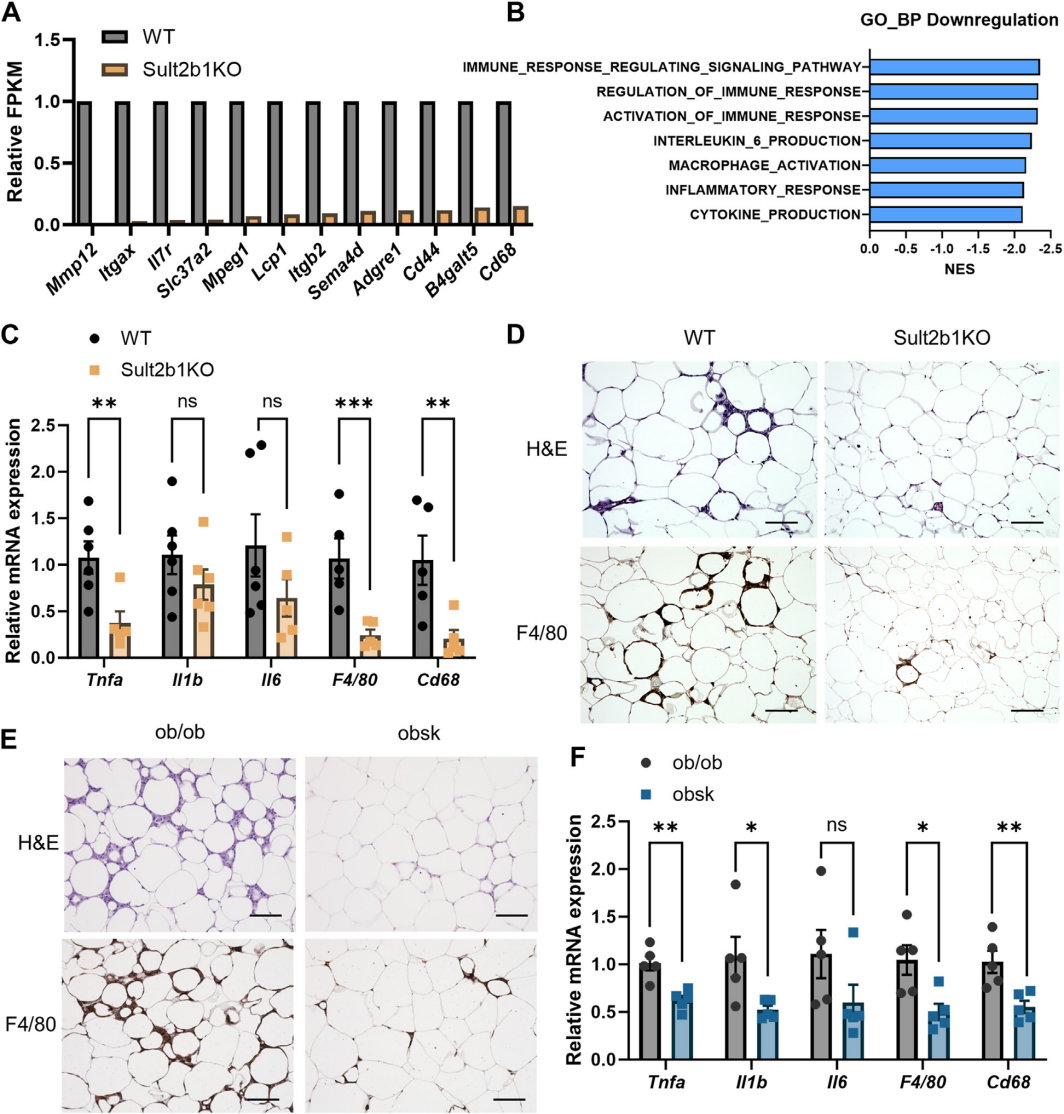
**Sult2b1 deficiency attenuates adipose inflammation**. WT and Sult2b1KO mice and the HFD treatment were the same as described in Figure 1. *A*, the expression of inflammation-related genes in eWAT after HFD feeding as revealed by RNA-seq. n = 4. *B*, GO pathways enriched in downregulated genes in eWAT from Sult2b1KO mice vs WT mice. *p* < 0.001 for all pathways shown. *C*, relative mRNA levels of WAT inflammation-related genes in eWAT after HFD feeding as measured by real-time PCR. *D-E*, H&E staining and F4/80 IHC staining in eWAT of WT and Sult2b1KO mice after HFD feeding (*D*), and of ob/ob and obsk mice (*E*). Scale bar, 100 μm. *F*, relative mRNA levels of WAT inflammation-related genes in eWAT of ob/ob and obsk mice. Data is represented as mean ± SD. Significance analysis was performed with unpaired Student’s *t* test and multiple t tests. ∗*p* < 0.05; ∗∗*p* < 0.01; ∗∗∗*p* < 0.001. ns, no significance.

Obesity causes inflammation in adipose tissue by dysfunctional adipocytes and infiltration of immune cells (20). Adipose inflammation is considered a major contributor to worsened metabolic functions (21). Our initial RNA-seq results revealed decreased expression of inflammation marker genes in the eWAT of HFD-fed Sult2b1KO mice (Fig. 5*A*). GSEA analysis confirmed a dramatic decrease in inflammation- and immune response-related pathways (Fig. 5*B*), and the downregulation of inflammation marker genes was further verified by qPCR analysis (Fig. 5*C*). Among the significantly decreased inflammation marker genes, the change in macrophage-related genes was the most prominent, which was consistent with the notion that macrophage infiltration, including the formation of crown-like structures, is the most well-established immune cell response in white adipose tissue in metabolic disease (22). At the histological level, the WAT of Sult2b1KO mice showed less macrophage infiltration as suggested by less abundance of crown-like structures based on the immunohistochemical staining of F4/80 (Fig. 5*D*). The obsk mice showed a similar decrease in the crown-like structures in their WAT (Fig. 5*E*), as well as the suppression of mRNA expression of the inflammation marker genes (Fig. 5*F*). These results suggested the attenuation of adipose inflammation may have also contributed to the metabolic benefits of Sult2b1 ablation.

### Sult2b1 knockout mice show improved energy expenditure and thermogenesis

**Figure 6:**
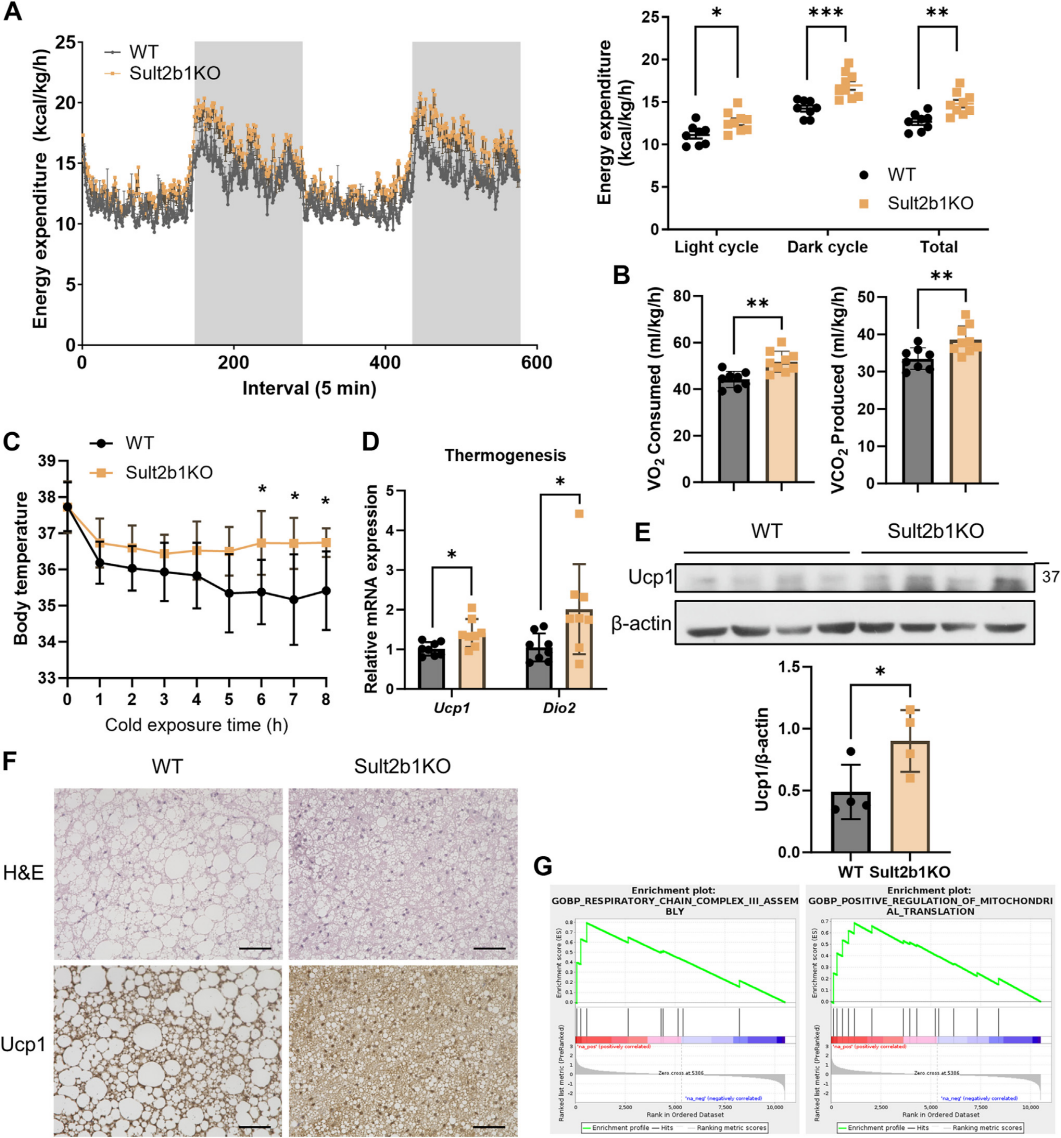
**Sult2b1 knockout mice show improved energy expenditure and thermogenesis.** WT and Sult2b1KO mice and the HFD treatment were the same as described in Figure 1. *A*, hourly energy expenditure for 48 h (left panel) and the average energy expenditure during the light and dark cycles (right panel) were determined. n = 8 to 9. *B*, average O_2_ consumption rate (upper panel) and CO_2_ production rate (*lower panel*) during 48 h monitor. n = 8 to 9. (*C*) Rectal temperature during 8 h cold exposure challenge after 12 weeks of HFD feeding. n = 9. *D*, relative mRNA levels of thermogenesis genes in BAT after 12 weeks of HFD feeding. *E*, Ucp1 protein levels in BAT determined by Western blotting. β-actin was used as a loading control. Quantification of the ratio of Ucp1 to β-actin is shown by bar graph. n = 4. *F*, H&E staining and Ucp1 IHC staining in BAT of WT and Sult2b1KO mice after HFD feeding. Scale bar, 100 μm. *G*, GSEA enrichment analysis of RNA-seq data derived from BAT of HFD-fed mice. Sult2b1KO vs. WT. Data are represented as mean ± SD. Significance analysis was performed with unpaired Student’s *t* test. ∗*p* < 0.05; ∗∗*p* < 0.01; ∗∗∗*p* < 0.001.

Energy expenditure and thermogenesis also play a key role in energy homeostasis. Specifically, higher energy expenditure can tilt the energy balance to the negative side and reduce weight gain when the energy intake is unchanged (23, 24). To investigate the effect of Sult2b1 ablation on energy expenditure, we conducted a metabolic cage study after 12 weeks of HFD feeding. Our results showed that the HFD-fed Sult2b1KO mice consumed more energy than their WT counterparts during both the light cycle and the dark cycle (Fig. 6*A*), while mice of both genotypes had comparable food intake (Fig. S5*A*) and locomotive activity (Fig. S5*B*). Consistently, oxygen consumption and carbon dioxide production were also significantly increased in the Sult2b1KO mice (Fig. 6*B*). Compared to the ob/ob mice, the obsk mice also showed increased energy expenditure (Fig. S5*C*) without affecting their locomotive activity (Fig. S5*D*).

Thermogenesis is a key metabolic process to dissipate energy while producing heat (25). To evaluate the thermogenesis of Sult2b1KO mice, we cold-challenged the mice at 4 °C for 8 h following a 12-week HFD feeding. Compared to their WT counterparts, the HFD-fed Sult2b1KO mice were more efficient in maintaining their body temperatures, as evidenced by the significantly higher rectal temperatures starting from 6 h of cold exposure (Fig. 6*C*). BAT plays an essential role in thermogenesis. Consistently, the mRNA expression of BAT genes involved in thermogenesis, including uncoupled protein 1 (*Ucp1*) and iodothyronine deiodinase 2 (*Dio2*), were elevated in Sult2b1KO mice (Fig. 6*D*). The induction of UCP1 protein in the BAT of Sult2b1KO mice was verified by both Western blotting (Fig. 6*E*) and IHC staining (Fig. 6*F*). Histological staining also showed less and smaller fat droplets in the BAT of Sult2b1KO mice (Fig. 6*F*). GSEA analysis of BAT RNA-seq results revealed increased mitochondrial activity in Sult2b1KO mice (Fig. 6*G*). Collectively, these results indicated that the ablation of Sult2b1 improves energy expenditure and thermogenesis in BAT, which likely has contributed to the protection of mice from weight gain and metabolic dysfunction.

### Sult2b1 ablation decreases serum fatty acid species and inhibits intestinal lipid uptake

**Figure 7:**
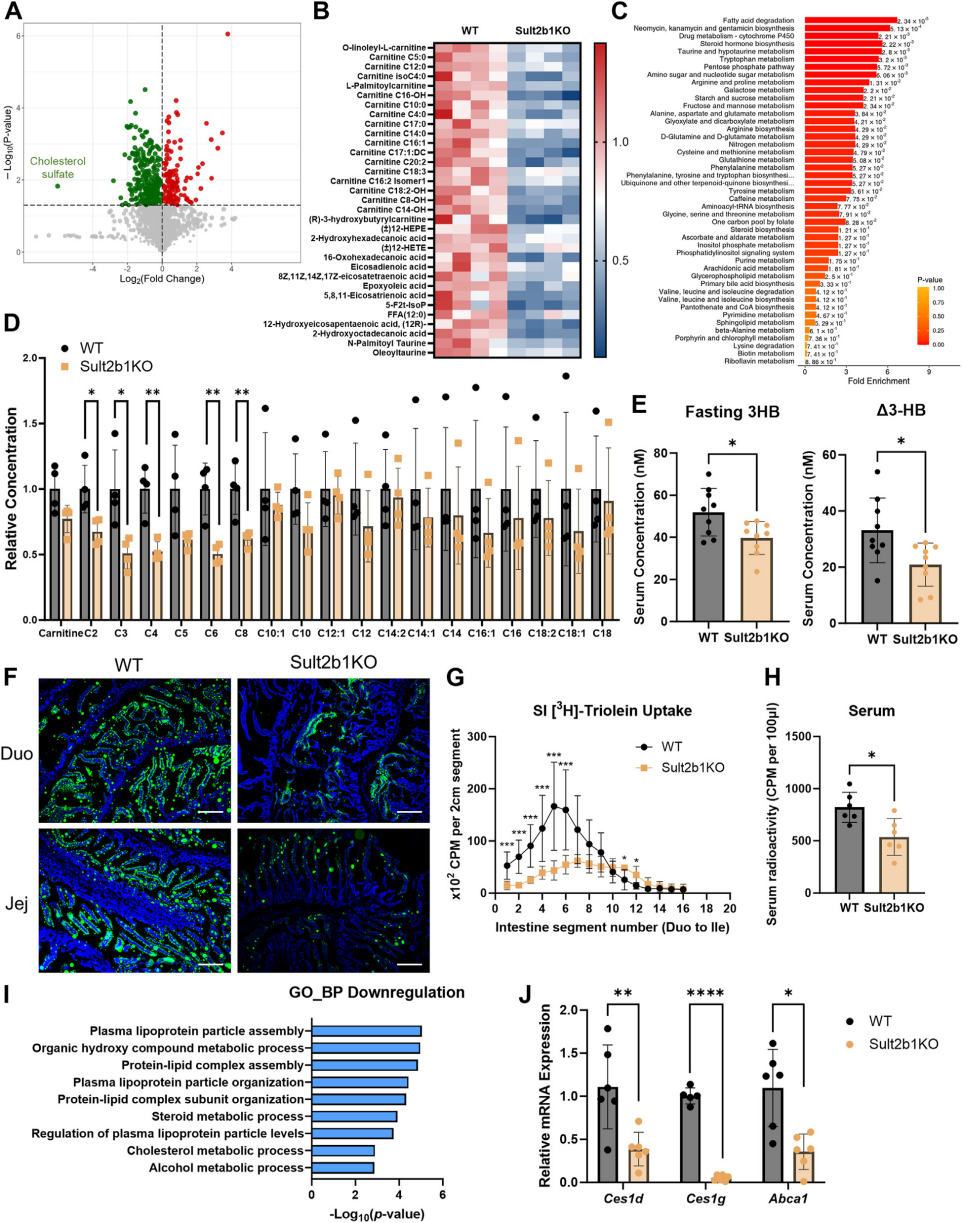
**Sult2b1 ablation decreases serum fatty acid species and inhibits intestinal lipid uptake**. *A*, Volcano plot showing differential metabolites from serum metabolomic analysis derived from HFD-fed WT and Sult2b1KO mice. Cholesterol sulfate is labeled. *B*, heatmap of fatty acids among differential serum metabolites. n = 4. *C*, top 50 metabolic sets by MSEA enrichment analysis. Sult2b1KO vs. WT. *D*, serum carnitines levels were measured by LC-MS/MS. Concentrations were normalized to levels in the WT group. n = 4. *E*, production of 3-HB after overnight fasting. Δ3HB = Fasting 3HB – Fed 3HB. n = 9. *F*, fluorescence visualization of lipid uptake in duodenum and jejunum 2 h after an oral gavage of olive oil containing BODIPY-labeled FA. Scale bar, 100 μm. *G-H*, distribution of radioactivity in small intestinal segments (*G*) and serum (*H*) 2 h after an oral challenge of olive oil containing [^3^H]-triolein. n = 6. *I*, GO pathways that enriched in downregulated genes in the jejunum. Sult2b1KO mice vs WT mice. *J*, relative mRNA levels of *Ces1d*, *Ces1g*, and *Abca1* in the jejunum after HFD feeding. n = 6. Data is represented as mean ± SD. Significance analysis was performed with unpaired Student’s *t* test and multiple *t*-tests. ∗*p* < 0.05; ∗∗*p* < 0.01; ∗∗∗*p* < 0.001; ∗∗∗∗*p* < 0.0001.

The pleiotropic metabolic benefits observed in HFD-fed Sult2b1KO mice prompted us to perform serum metabolic analysis to determine whether there are circulating factors that may have accounted for the systemic benefits. Metabolomic analysis on the serum of HFD-fed mice showed that the serum level of cholesterol sulfate was dramatically decreased in Sult2b1KO mice as expected (Fig. 7*A*). Further analysis of the metabolomic results showed the serum level of fatty acid species was significantly downregulated by Sult2b1 ablation (Figs. 7*B* and S6*A*). The downregulated metabolites in Sult2b1KO mice were mostly related to fatty acid degradation pathway (Fig. 7*C*). Among the downregulated fatty acids, many of them were acyl-carnitines, the intermediates of fatty acid oxidation. The decreased levels of acyl-carnitines in HFD-fed Sult2b1KO mice were further verified by LC-MS analysis (Fig. 7*D*). We speculated that fatty acid oxidation was decreased by Sult2b1 ablation, because both the hepatic expression of *Cpt1a* gene (Fig. 3*F*) and acyl-carnitine levels were downregulated in Sult2b1KO mice. To further evaluate fatty acid oxidation *in vivo*, we compared the level of 3-hydroxybutyrate (3HB) after overnight fasting, which is a major product of fatty acid oxidation during fasting (26). Sult2b1KO mice produced less 3HB than WT mice (Fig. 7*E*), further suggesting that fatty acid metabolism was decreased in Sult2b1KO mice.

Since fatty acid levels and their metabolism were both decreased in Sult2b1KO mice, we then speculated that the intestinal dietary fatty acid uptake may have been inhibited by Sult2b1 deficiency. When the expression of Sult2b1 in the small intestines was evaluated, we found that compared to other metabolic tissues, Sult2b1 was highly expressed in the small intestines (Fig. S6*B*), although the intestinal expression of Sult2b1 was not regulated by HFD-feeding (Fig. S6*C*). To directly visualize intestinal fatty acid uptake into the enterocytes, we administered BODIPY-labeled fatty acid to mice *via* oral gavage. While we observed abundant fluorescence signals in the duodenum and jejunum of the WT mice and ob/ob mice as expected, the intestinal fluorescence signals were dramatically reduced in Sult2b1KO mice (Fig. 7*F*) and obsk mice (Fig. S6*D*). The inhibition of intestinal fatty acid uptake in Sult2b1KO mice was further verified by radiolabeled fatty acid uptake analysis, as the Sult2b1KO mice exhibited reduced maximum [^3^H]-triolein uptake in the jejunum and a shift in uptake to more distal segments (Fig. 7*G*). The decrease of serum level of [^3^H]-triolein was also observed in Sult2b1KO mice, consistent with their inhibition of intestinal lipid uptake (Fig. 7*H*).

To understand the inhibition of intestinal lipid uptake by Sult2b1 ablation, we first evaluated the expression of intestinal fatty acid transporters *Cd36*, fatty acid transport protein (*Fatp4*), and plasma membrane fatty acid-binding protein (*Fabppm*) that are known to facilitate lipid absorption in the small intestines. However, the expression of none of these genes was decreased in either duodenum or jejunum (Fig. S6*E*). Besides fatty acid transporter, lipoproteins are also key players in dietary lipid absorption by the small intestines, because lipids uptaken by enterocytes are packaged into lipoprotein for transportation into the circulation (27, 28). Indeed, our GSEA analysis of jejunum RNA-sequencing results indicates that several lipoprotein particle assembly pathways were significantly downregulated in Sult2b1KO mice (Fig. 7*I*). The downregulation of several common genes that belong to these pathways, such as *Ces1d*, *Ces1g* and *Abca1*, was validated by qPCR (Fig. 7*I*). The carboxylesterase 1 (*Ces1*) genes are known to regulate lipoprotein assembly (29), whereas *Abca1* is a lipid transporter that plays a key role in the production of high-density lipoprotein (HDL) (30). Taken together, our results demonstrated that the ablation of Sult2b1 inhibits intestinal lipid absorption potentially by interfering with lipoprotein assembly, which leads to the decrease of systemic fatty acid level and ameliorates obesity.

## Discussion

SULT2B1 was previously known to decrease serum and hepatic lipid levels by deactivating the oxysteroid-derivative endogenous LXR agonists (14), and inhibit gluconeogenesis by targeting HNF4α (15), both of which may have contributed to the alleviation of metabolic abnormalities in obesity. As such, we initially hypothesized that the loss of Sult2b1 would lead to worsened metabolic phenotype in mice challenged with HFD or in ob/ob mice. Surprisingly, our data showed that Sult2b1KO mice were also protected from obesity and associated metabolic abnormalities, including body weight gain and fat accumulation, hyperlipidemia, insulin resistance, hepatic steatosis, and adipose tissue inflammation.

A potential explanation for the counterintuitive observations is the tissue-specific effect of Sult2b1. In our previous gain-of-function study, the transgenic effect of Sult2b1 was primarily focused on the liver because the transgene was targeted to the liver (15). In the current study, the Sult2b1KO mouse is a global loss-of-function model that includes the loss of Sult2b1 in tissues other than the liver, leading to an overall protective phenotype. Our results showed that liver-specific reconstitution of Sult2b1 failed to abolish the metabolic benefit in the Sult2b1KO mice, suggesting that it is unlikely that the loss of Sult2b1 in the liver can solely account for the metabolic benefit in the whole-body Sult2b1KO mice. Instead, the loss of Sult2b1 in extrahepatic tissues is likely responsible for the metabolic benefit. More tissue-specific investigations in the future, including the creation and use of tissue-specific Sult2b1 knockout mice, are necessary to pinpoint the tissues or cell types that are responsible for the role of Sult2b1 in the development of obesity and insulin resistance. In the liver, we also cannot exclude the possibility that the compensatory regulation of other SULT isoforms may have contributed to the phenotypical exhibition.

The adipose tissue-specific improvement of insulin sensitivity in the Sult2b1KO mice was intriguing. The HFD-fed Sult2b1KO mice showed a better ITT performance. Interestingly, upon insulin injection, the insulin signaling as indicated by the phosphorylation of AKT was only upregulated in the WAT and BAT, but not the liver or skeletal muscle of Sult2b1KO mice. In patients with obesity, insulin resistance can be triggered by WAT inflammation (31), as WAT inflammation leads to the release of pro-inflammatory cytokines, such as TNFα, IL-1β, IL-6, that inhibit insulin signaling and impaired glucose uptake (32). WAT inflammation was ameliorated in our HFD-fed Sult2b1KO mice and obsk mice, as indicated by their decreased crown-like structures and decreased expression of pro-inflammatory cytokines, which likely has contributed to the improved insulin sensitivity in this tissue.

Another evidence for the adipose tissue contrition to the phenotypic exhibition is the effect of Sult2b1KO ablation on thermogenesis, likely mediated by BAT. Our metabolic cage study showed Sult2b1KO mice had greater energy expenditure than their WT counterparts without affecting locomotive activity and food intake. Elevated thermogenesis in Sult2b1KO mice was further supported by gene expression analysis and cold exposure challenge. BAT, as the major energy expenditure regulator tissue, showed increased thermogenesis and mitochondrial activity upon the loss of Sult2b1. It remains to be determined whether the increased thermogenesis resulted directly from the loss of Sult2b1 in BAT, or it is secondary to other local or systemic metabolic changes. For instance, our serum metabolomic analysis showed the adenosine level was dramatically increased in Sult2b1KO mice (data not shown). Since adenosine has been reported to counteract obesity by activating brown adipocytes (33, 34), the elevated circulating level of adenosine may have contributed to the increased BAT thermogenesis. Future studies are necessary to define the metabolic effect of SULT2B1 in WAT and BAT.

Another interesting finding is the inhibition of intestinal dietary lipid absorption in Sult2b1KO mice, which was initially suggested by the concurrent decrease of fatty acid levels in the serum and suppression of fatty acid oxidation. We speculated that dietary lipid absorption was inhibited, which led to both lower fatty acid level and lower fatty acid metabolism. Indeed, both fluorescence- and isotope-labeled fatty acid uptake assays demonstrated a decreased intestinal lipid absorption in Sult2b1KO mice. Interestingly, the major intestinal fatty acid transporters did not show significant changes in Sult2b1KO mice. Instead, the lipoprotein particle assembly-related pathways and genes were downregulated. Lipids, after being uptaken by enterocytes, are packaged into lipoprotein for transportation into the circulation (27, 28). Among genes downregulated in the Sult2b1KO small intestine, Ces1d facilitates the very-low density lipoprotein (VLDL) assembly in the liver and chylomicron secretion. Knockout of Ces1d protects mice from hyperlipidemia (29). Similarly, ablation of Ces1g reduced cholesterol and triglyceride absorption in an atherosclerosis model (35). We reason the downregulation of Ces1d and Ces1g may have contributed to the inhibition of intestinal lipid absorption, but the direct link between intestinal Sult2b1 ablation and suppression of Ces1d and Ces1g warrants more studies. Based on the human protein atlas and our mouse data, the expression of SULT2B1 in humans and rodents is higher in the small intestines than several other metabolic tissues, including the liver, adipose tissue, and skeletal muscle (Fig. S6*B*), although the small intestine expression of Sult2b1 was not regulated by HFD feeding (Fig. S6*C*), suggesting that the intestinal SULT2B1 may play the dominant role in obesity and the related metabolic syndromes. A limitation of this study is that the use of whole-body knockout mice prevented us from concluding the tissue-specific effect of loss of Sult2b1. Future creation and use of intestine-specific Sult2b1 knockout mice will help to unveil the intestinal function of SULT2B1.

Obesity is increasingly a worldwide health concern. Most of the FDA-approved drugs for weight management target the central nervous system, such as glucagon-like peptide-1 receptor (GLP-1R) and melanocortin-4 receptor (MC4R) pathway, to regulate appetite and satiety (7). Given the limited options, finding new drug targets remains essential to combat the epidemic of obesity and metabolic syndrome. Our results suggested that inhibition of SULT2B1 may be explored for managing obesity and insulin resistance by increasing energy expenditure and inhibiting intestinal fat absorption.

## Experimental procedures

### Animal models

Sult2b1 whole-body knockout (Sult2b1KO) and ob/ob mice were purchased from The Jackson Laboratory (Strain #:018,773) and bred in-house. Sult2b1 whole-body knockout ob/ob mice were generated by crossing the ob/ob mice with Sult2b1KO mice. Liver-specific Cd36 transgenic (CD36 Tg) mice were described by us before (17). Cd36 liver-overexpressed Sult2b1KO mice were generated by crossing the CD36 Tg mice with Sult2b1KO mice. For the diet-induced obesity (DIO) model, 8-week-old male mice were fed a high-fat diet (HFD) (60% fat; Research Diets, D124924) for 12 or 16 weeks as indicated. Animal body composition, including the fat mass and lean mass, was evaluated weekly using the EchoMRI. All mice were housed *ad libitum* on a 12-h/12-h light/dark cycle under pathogen-free conditions. All experimental procedures were performed in accordance with relevant federal guidelines and with the approval of the University of Pittsburgh Institutional Animal Care and Use Committee (IACUC).

### Insulin tolerance test, glucose tolerance test, and homeostatic model assessment

ITT and GTT were performed 8 weeks after the start of HFD-feeding or at 13 weeks old for ob/ob mice. For GTT, after a 16h overnight fasting, glucose (Sigma) was given at 2 g/kg body weight by intraperitoneal (i.p.) injection. For ITT, after a 6-h fasting, mice were given an i.p. injection of 0.50 U/kg bodyweight insulin (Humulin N, Eli Lilly). Blood glucose was monitored right before injection and every 30 min following injection for 2 h using a glucometer. Homeostatic model assessment for insulin resistance (HOMA-IR) were calculated using the following formulas: insulin resistance = [fasting insulin (μU/ml)] ∗ [fasting glucose level (mg/dl)]/405, as previously described (36).

### Acute insulin sensitivity test

Mice that received 12 weeks of HFD feeding were treated with 0.75 U/kg insulin by i.p. injection after 6 h fasting. Liver, eWAT, BAT, and soleus were collected 17 min after the insulin injection.

### Liver and serum biochemical analysis

Blood was collected *via* cardiac puncture and subsequently centrifuged twice at 8000×*g* for 5 min to collect the serum. The serum levels of total triglyceride cholesterol, ALT, AST (Stanbio Laboratory), and insulin (Crystal Chem) were measured using commercial assay kits. Liver lipids were extracted using the chloroform/methanol Folch method as described previously (37) before determination of triglyceride levels.

### Histology, immunohistochemistry (IHC), and immunofluorescence (IF) staining

After harvesting, tissues were fixed in 10% neutral buffered formalin for 24 h, and then dehydrated, embedded in paraffin, and sectioned at 4 μm. The general histology was evaluated by hematoxylin and eosin (H&E) staining. For the IHC or IF staining of Cd36, F4/80, and Ucp1, antigen retrieval was performed by heat-mediated epitope retrieval in citric acid solution (pH 6.0) at 94 °C for 20 min. The primary antibodies of Cd36, F4/80, Ucp1, and the secondary antibody of Cd36 IF staining are listed in Table S1. All the antibodies were previously verified or stained with positive control. The Vectastain ABC Kit (PK-6101) and DAB Peroxidase Substrate Kit (SK-4105) from Vector Laboratories were used for IHC staining.

### Oil Red O staining

Liver tissues were fixed in 1% paraformaldehyde (PFA) for 3 h at 4 °C followed by overnight dehydration in 30% sucrose solution and embedded in O.C.T compound. The frozen tissues were sectioned using Cryostat at 8 microns and stained with Oil Red O (0.5% in isopropanol) for 15 min.

### Metabolic cage study

Following 12 weeks of HFD-feeding, mice were placed in metabolic cages (Columbus Labs Animal Monitoring System) for 72 h. Mice were individually housed and feeding, activity, and energy expenditure were tracked for 48 h. Following standard procedure (38), data was not recorded for the first 24 h as this is an acclimation period for the mice to the new environment. 12-h light/dark cycles were simulated and free access to HFD was provided. The reported indirect calorimetry was calculated using the Weir equation (39), which determines energy expenditure from the consumption of O_2_ and the production of CO_2_.

### Cold exposure

Mice were single-housed and placed in precooled cages at 4 °C with bedding and free access to chow and water. Core body temperatures were recorded using Oakton Acorn Temp JKT thermocouple meter with a rectal probe every 60 min.

### Untargeted serum metabolomic analysis

This was custom conducted at BGI. In brief, 50 μl of the serum sample and 300 μl of the extraction solution (ACN: MeOH = 1:4, v/v) containing internal standards were mixed and vortexed for 3 min, then centrifuged at 12,000 rpm for 10 min (4 °C). Two hundred μL of the supernatant was collected and placed at −20 °C for 30 min, followed by centrifugation at 12,000 rpm for 3 min (4 °C). A 180 μl aliquot of the supernatant was used for LC-MS/MS analysis using an ExionLC AD UHPLC coupled to a TripleTOF 6600+ mass spectrometer (Sciex). The combination of T3 (Waters ACQUITY UPLC HSS T3 1.8 μm, 2.1 mm∗100 mm) and HILIC (ACQUITY Premier BEH Amide 1.7 μm, 2.1 mm∗150 mm) both at a column temperature of 40 °C was applied to expand the metabolome coverage. For T3 column, Solvent A was ultrapure water (0.1% formic acid added), and solvent B was acetonitrile (0.1% formic acid added). The gradient was set as follows: 5% B at 0 min, 20% B at 2 min, 60% B at 5 min, 99% B at 6 min, 99% B at 7.5 min, 5% B at 7.6 min, and 5% B at 10 min. The flow rate was set to 0.4 ml/min. For HILIC column, Solvent A was 60% ACN, 30% water, and 10% MeOH with 20 mM ammonium formate, pH 10.6, and solvent B was 40% ACN and 60% water with 20 mM ammonium formate, pH 10.6. The gradient was set as follows: 5% B at 0 min, 30% B at 3.5 min, 95% B at 5.5 min, 95% B at 6.5 min, 5% B at 6.51 min, and 5% B at 10 min. The flow rate was set to 0.4 ml/min. The mass spectrum conditions were as follows: ion spray voltage, 5000 V (ESI+) and −4000 V (ESI-); heater temperature, 550 °C (ESI+) and 450 °C (ESI-); source gas 1, 50 psi; source gas 2, 60 psi; curtain gas, 35 psi; MS1 collision energy, 10V(ESI+) and −10V (ESI-); MS2 collision energy 30V (ESI+) and −30V (ESI−); and collision energy spread, 15V. The mass scanning range was set at 50–1000 m/z (MS1) and 25–1000 m/z (MS2). Solvents used for extractions and LC-MS/MS analysis were of UPLC grade or better from Fisher Scientific (Fairlawn, NJ) or Sigma (St Louis, MO).

### Sample preparation and derivatization for carnitine LC-MS/MS quantification

Serum (10 μl) was precipitated with 100 μl of cold methanol containing an internal standard mix composed of ^2^H_9_-carnitine (49 ng), ^2^H_3_-Acetylcarnitine(15.4 ng), ^2^H_3_-Propionylcarnitine (3.3 ng), ^2^H_3_-Butyrylcarnitine (3.51 ng), ^2^H_9_-Isovalerylcarnitine (3.72 ng), ^2^H_3_-Octanoylcarnitine (4.36 ng), ^2^H_9_-Myristoylcarnitine (5.64 ng), and ^2^H_9_-Palmitoylcarnitine (12.06 ng) (Cambridge Isotopes Laboratories Inc.). Samples were vortexed, centrifuged, and the supernatant evaporated under nitrogen. Acylcarnitines were derivatized to their butyl esters, as previously reported (40). Briefly, 100 μl of n-butanol containing 5% v/v acetyl chloride was added to the dried samples, incubated at 60 °C for 20 min, and then evaporated under nitrogen. The samples were reconstituted in 500 μl of methanol for mass spectrometry analysis. Solvents used for extractions and mass spectrometry analyses were of HPLC grade or better from Fisher Scientific.

Acyl-carnitine quantification was performed in multiple reaction monitoring (MRM) mode using a QTrap 6500+ triple quadrupole mass spectrometer (Sciex) equipped with an electrospray ionization (ESI) source operating in positive mode. The source settings were as follows: ion spray voltage, 5500 V; heater temperature, 450°C; source gas 1, 65 psi; source gas 2, 75 psi; curtain gas, 30 psi; and collision gas (CAD), medium. Chromatographic separation was achieved on a C8 column (2 mm × 150 mm, 5 μm, Phenomenex) at a column temperature of 40 °C. Solvent A consisted of 0.1% formic acid, and solvent B consisted of a mixture of isopropanol/acetonitrile (20/80, v/v) with 0.1% formic acid. Gradient elution was performed with the following program: 5% B (0.5 ml/min) for 0.4 min, then a linear increase to 100% B over 11.6 min, a hold at 100% B for 3 min, followed by a re-equilibration step at 5% B for 4 min. Analytes were measured in scheduled MRM mode following the charged loss of m/z 85.

### *In vivo* intestinal lipid absorption assays

The intestinal uptake of dietary fat was assessed as previously described (41). For fluorescence detection, mice were fasted for 4 h and orally gavaged with BODIPY 500/510 C1, C12 FAs (2 μg/g body weight; Invitrogen #D3823) in 100 μl olive oil. Small intestines were collected and frozen in optimum cutting temperature (O.C.T) compound 2 h after the gavage. 8 μm sections were cut, mounted with DAPI Fluoromount-G, and examined under a fluorescence microscope. For radiation detection, mice were fasted for 4 h and administered with 1 μCi of [^3^H]-Triolein in 200 μl olive oil by gavage. After 2 h, mouse blood was collected, and the small intestine was excised and flushed with 0.5 mM sodium taurocholate in PBS and cut into 2-cm segments. Segments were then digested with 500 μl of 1 N NaOH at 65 °C overnight and mixed with 5 ml of Hionicfluor liquid scintillation cocktail (Revvity). Scintillation was measured as counts per minute with a Beckman Coulter LS6500 Liquid Scintillation Counter.

### RNA sequencing analysis

Total RNA was extracted with the RNeasy Mini Kit (Qiagen) and purified with the RNA Clean & Concentrator Kit (Zymo Research). Library preparation and RNA-Seq were performed by the Health Sciences Sequencing Core at the Children’s Hospital of Pittsburgh or Novogene. RNA-Seq analysis was performed as we have described (42). Reads were mapped to the mouse reference genome GRCm38 using STAR (43). RSEM (44) was used for gene expression quantification and differential expression analysis were performed by Cuffdiff (44) comparing the Sult2b1 whole-body knockout liver/BAT/WAT sample and their WT control respectively. Gene set enrichment analysis (GSEA) (45) was done on genes ranked by their signed log-transformed Cuffdiff *p*-values in GO (46) and KEGG (47). *p*-value <0.05 is used to show significantly upregulated/downregulated pathways.

### Quantitative real-time polymerase chain reaction

Tissue was homogenized and RNA was extracted using TRIzol reagent through phenol-chloroform extraction. Complementary DNA (cDNA) was reverse transcribed from 1 μg of total RNA using a cDNA reverse transcription kit (Fisher Scientific). SYBR Green-based real-time PCR was performed using the QuantStudio 6 Flex Real-Time PCR System. Data were normalized to Cyclophilin and Gapdh by the ΔΔCt method. PCR primer sequences are listed in Table S2.

### Western blotting

Tissues were homogenized and lysed in ice-cold RIPA buffer containing a protease inhibitor cocktail (Fisher Scientific). Pierce BCA Protein Assay Kit was used to assess protein concentration for sample preparation. 30 μg of protein extracts were separated on SDS-PAGE gels and transferred onto a polyvinylidene difluoride (PVDF) membrane. The primary antibodies of phosphorylated Akt, Akt, Cd36, β-actin, Sult2b1, Ucp1, and horseradish peroxidase (HRP)-conjugated secondary antibodies are listed in. Table S1. All the antibodies were previously verified or ran with a positive control. Bands were visualized using HRP-based Pierce ECL Western Blot Substrate or SuperSignal West Pico PLUS Chemiluminescent Substrate (Fisher Scientific). The densitometric analysis of the Western blot was performed using ImageJ.

### Statistical analysis

Data was presented as mean ± standard deviation (SD). All the individual data points are independent biological replicates. Statistical analysis between groups was conducted by Student’s *t* test, multiple *t*-tests, or one-way ANOVA with a *p*-value of <0.05 considered significant. Data conducted over intervals (*i*.*e*. MRI/Body weight/ITT/GTT) was analyzed by Student's *t* test at each interval. Data was compiled and presented using GraphPad Prism 8.

## Data availability

All data will be made available on request.

## Supporting information

This article contains supporting information.

## Conflict of interest

The authors declare that they have no conflicts of interest with the contents of this article.
