# Supplementary material for: Inhibition of sulfotransferase SULT2B1 prevents obesity and insulin resistance by regulating energy expenditure and intestinal lipid absorption
Source: J Biol Chem. 2025 May 31;301(7):110327. doi: 10.1016/j.jbc.2025.110327 (PMC12268192; doi:10.1016/j.jbc.2025.110327)
Supplement: Supporting Materials [file mmc1.docx]

**Figure S1. Characterization of Sult2b1 KO mice fed with control diet**

**
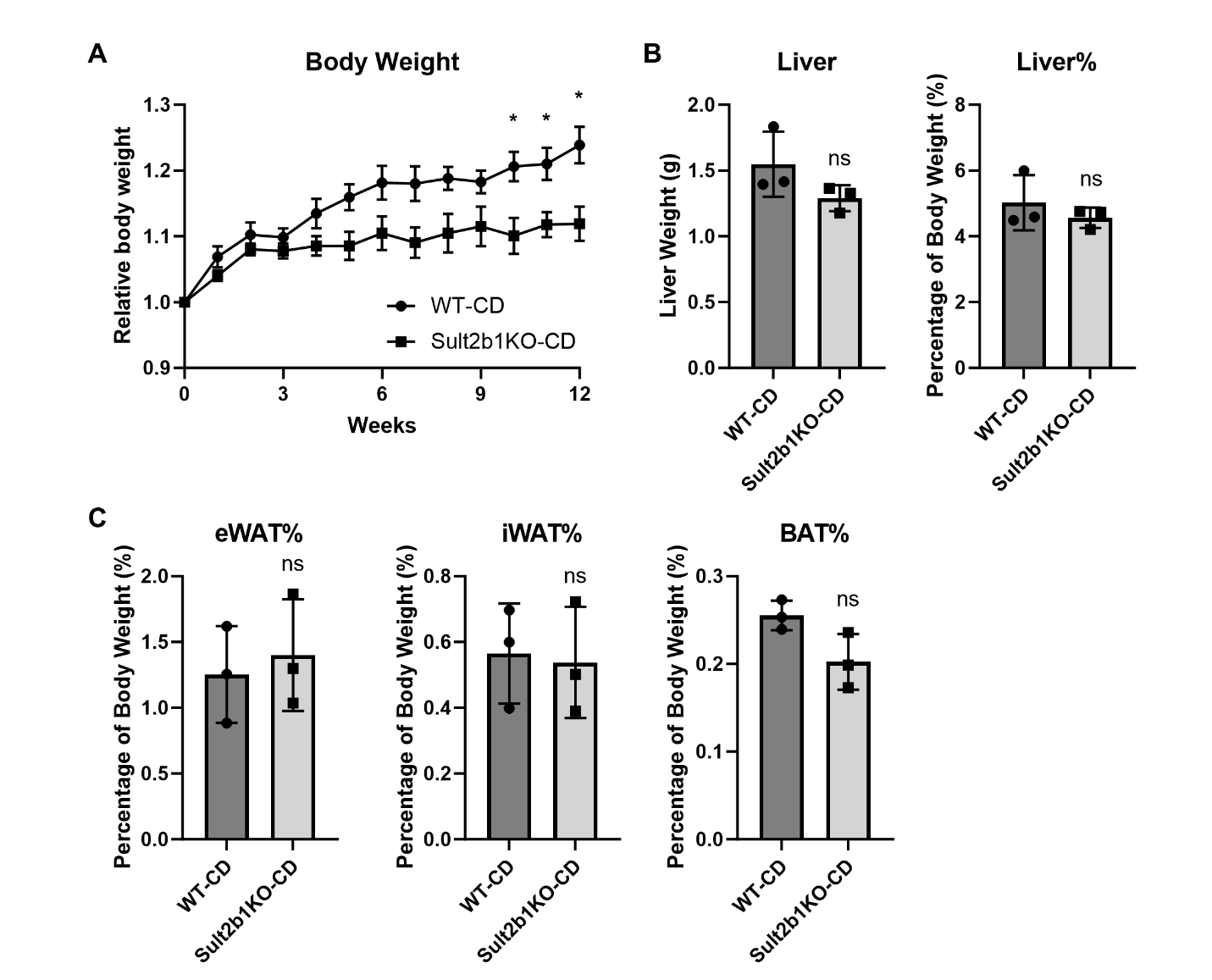
**

**Figure S2. Loss of Sult2b1 in ob/ob mice ameliorates obesity and hyperlipidemia
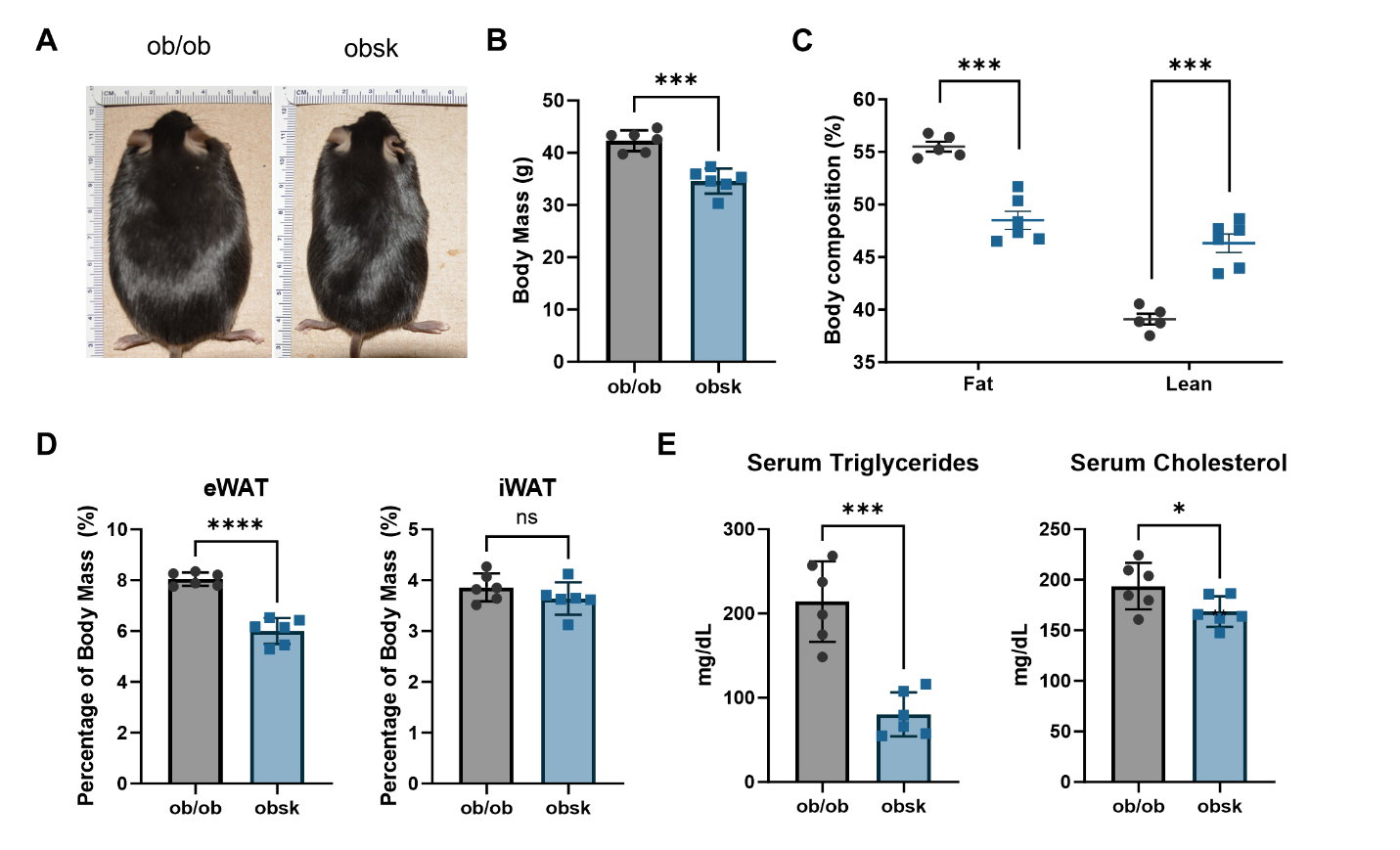
**

**Figure S3. Loss of Sult2b1 in ob/ob mice ameliorates hepatic steatosis**

**
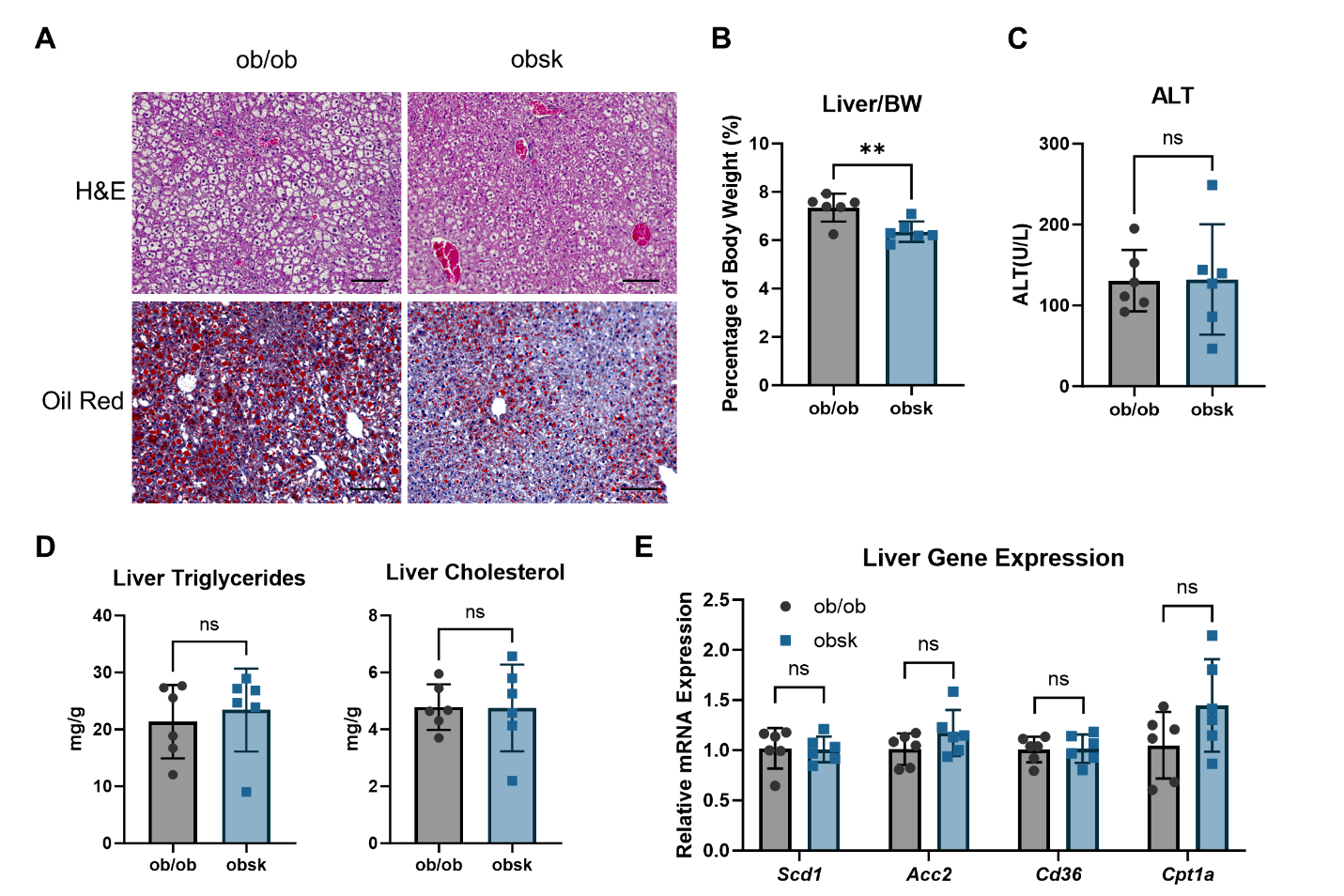
**

**Figure S4. The metabolic benefit of Sult2b1 ablation is independent of the suppression of hepatic Cd36**

**
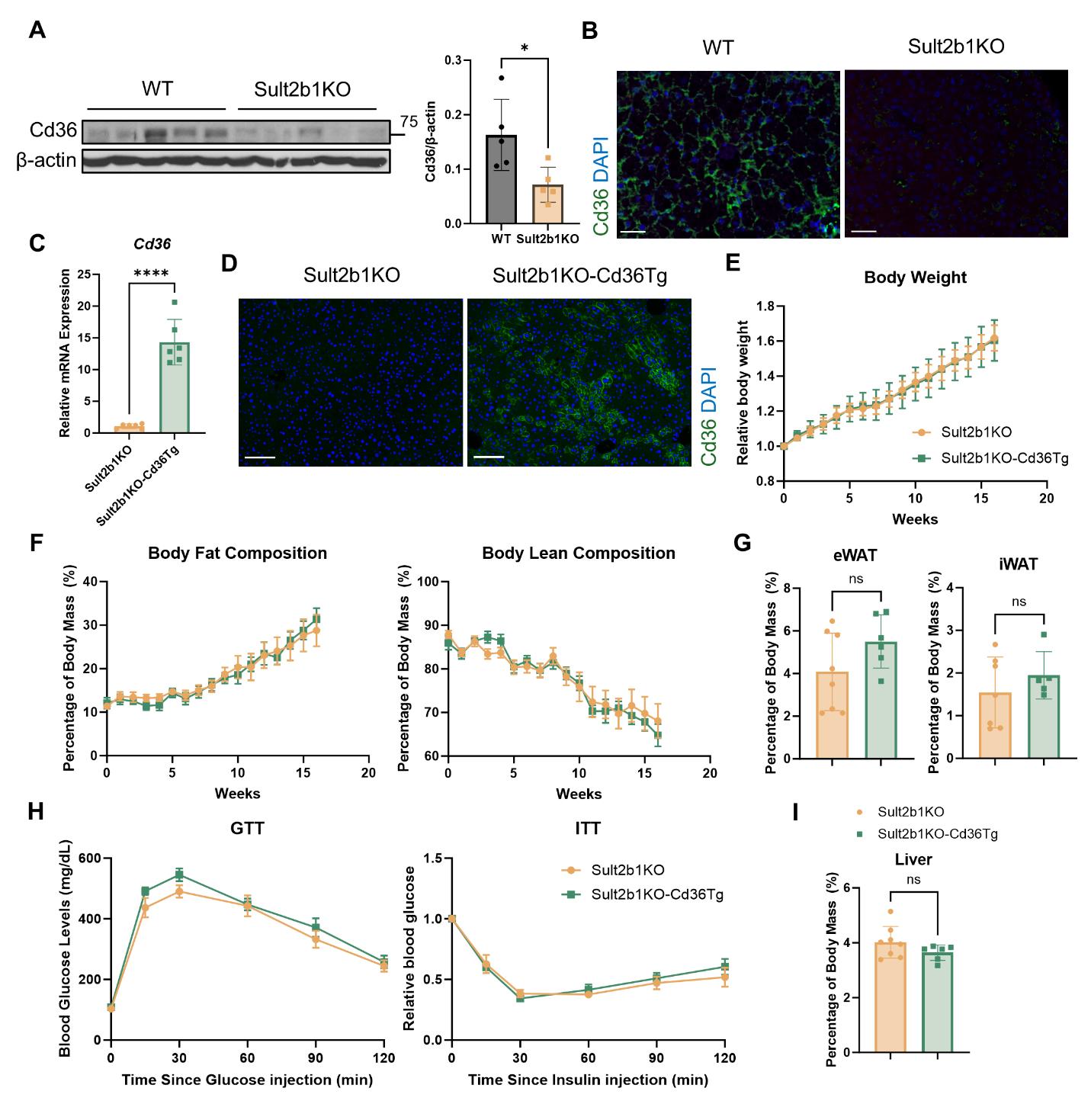
**

**Figure S5. Sult2b1 knockout mice show improved energy expenditure**

**
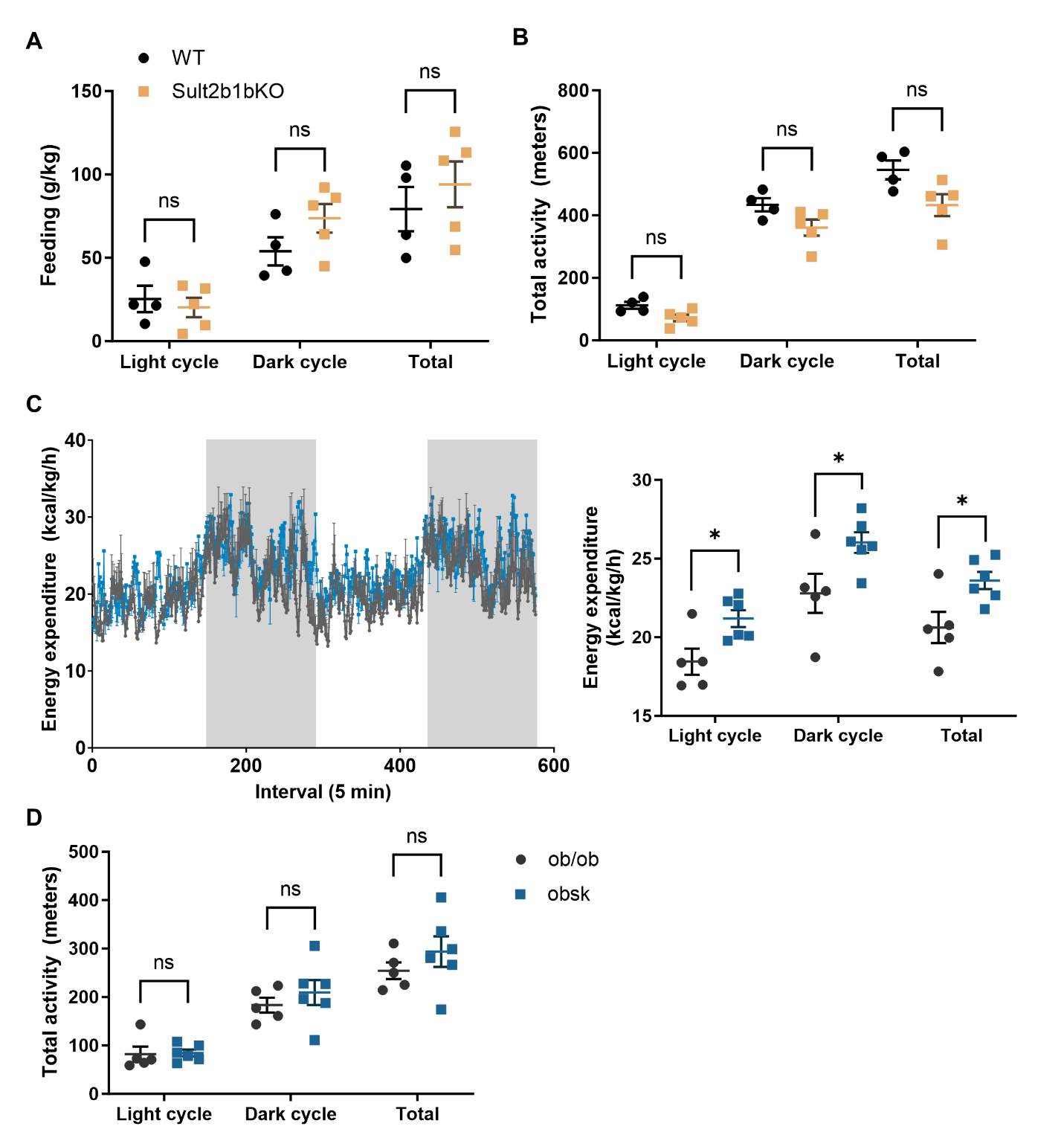
**

**Figure S6. Sult2b1 ablation decreases serum fatty acid species and inhibits intestinal lipid uptake**

**
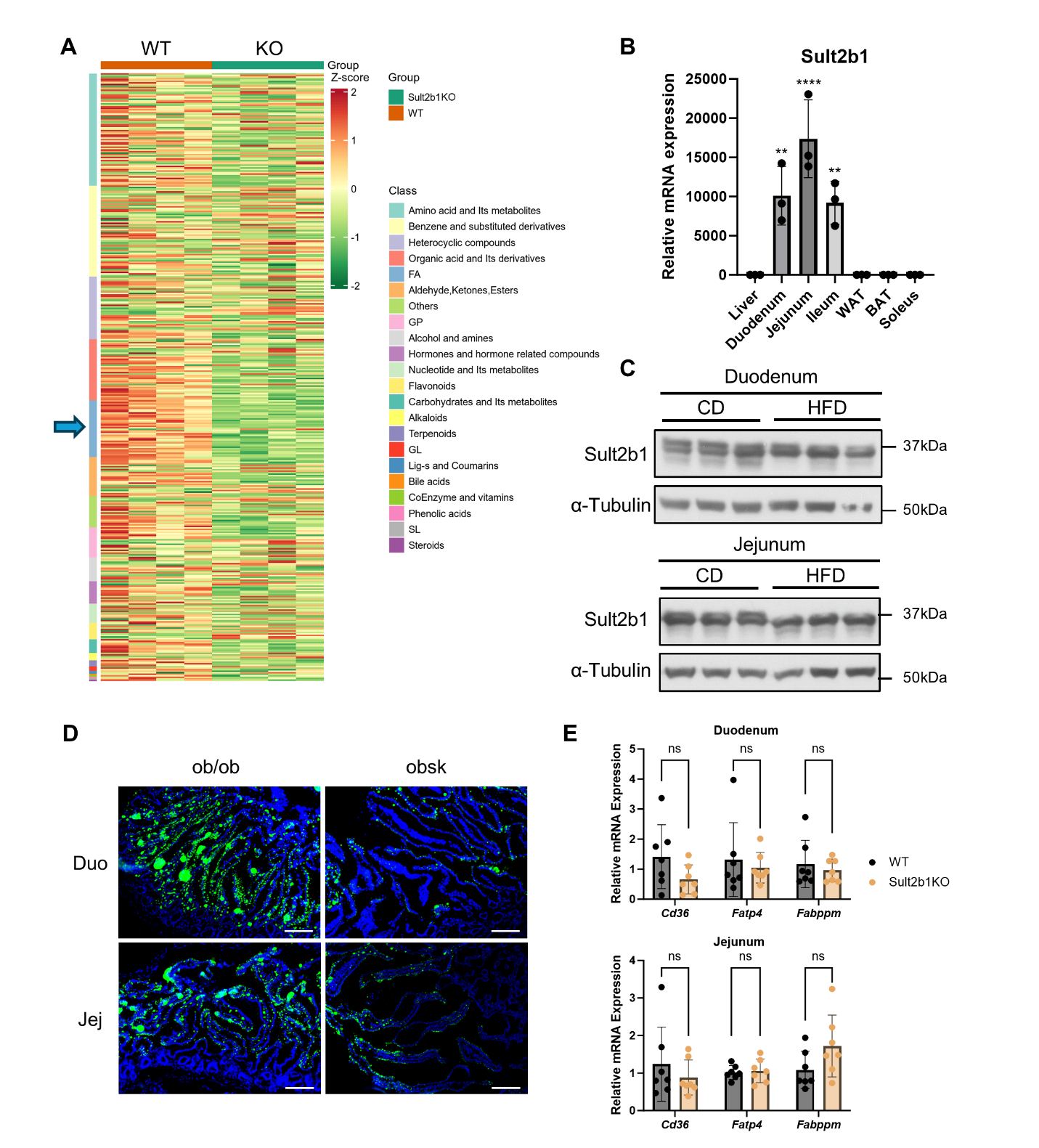
**

**Supplementary Figure Legends**

**Figure S1. Characterization of Sult2b1 KO mice fed with control diet**

Male WT and Sult2b1KO mice were fed with control chow diet until 20 weeks old. CD, control diet. n=3. **(A)** Time course of relative body weight. **(B)** Liver weight and relative liver weight at 20 weeks old. **(C)** Weights of eWAT, iWAT, and BAT as percentages of body weight at 20 weeks old. Data is represented as mean ± SD. Significance analysis was performed with unpaired Student’s *t*-test. *P < 0.05. ns, no significance.

**Figure S2. Loss of Sult2b1 in ob/ob mice ameliorates obesity and hyperlipidemia**

Thirteen-week-old male ob/ob and Sult2b1KO-ob/ob (obsk) mice were analyzed. Shown are representative photographs of the mice (A), body weight (B), body compositions measured by Echo-MRI (C), weights of eWAT and iWAT as percentages of body weight (D), and serum triglyceride and cholesterol levels (E). n=6. Data is represented as mean ± SD. Significance analysis was performed with unpaired Student’s *t*-test. *P < 0.05; **P < 0.01; ***P < 0.001; ****P < 0.0001. ns, no significance.

**Figure S3. Loss of Sult2b1 in ob/ob mice ameliorates hepatic steatosis**

Eight weeks old male ob/ob and obsk mice were analyzed. (A) H&E staining and Oil Red O staining of liver sections. Scale bar, 100 µm. (B-D) Relative liver weight (B), serum ALT level (C), and hepatic triglycerides and cholesterol levels (D). (E) Relative mRNA expression of lipid metabolism-related genes in the liver. n=6. Data is represented as mean ± SD. Significance analysis was performed with unpaired Student’s *t*-test. **P < 0.01. ns, no significance.

**Figure S4. The metabolic benefit of Sult2b1 ablation is independent of the suppression of hepatic Cd36**

(A) Cd36 protein levels in the livers of WT and Sult2b1KO mice after 12 weeks of HFD feeding. β-actin was used as a loading control. Quantification of the ratio of Cd36 to β-actin is shown by bar graph. n=5. (B) Immunofluorescence staining of Cd36 in liver of WT and Sult2b1KO mice after 12 weeks of HFD feeding. Scale bar, 50 µm. (C) Relative mRNA expression of Cd36 in the livers of Sult2b1KO and Sult2b1KO-CD36Tg mice. n=6. (D) Immunofluorescence staining of Cd36 in liver of Sult2b1KO and Sult2b1KO-CD36Tg mice. Scale bar, 100 µm. (E-I) 8 weeks old male Sult2b1KO (n=8) and Sult2b1KO-CD36Tg (n=6) mice were fed with HFD for 16 weeks. Shown are body weight (E), body fat and lean mass composition (F), weights of eWAT and iWAT as percentages of body weight (G), GTT and ITT (H), and relative liver weight (I). Data is represented as mean ± SD. Significance analysis was performed with unpaired Student’s *t*-test. *P < 0.05; ****P < 0.0001. ns, no significance.

**Figure S5. Sult2b1 knockout mice show improved energy expenditure**

(A-B) Eight weeks old male WT and Sult2b1KO mice were fed with HFD for 12 weeks. Average food intake (A) and total locomotive activity (B) during the light and dark cycles were determined. n=8-9. (C) Hourly energy expenditure for 48 h (left panel) and the average energy expenditure during the light and dark cycles (right panel) of 13 weeks old ob/ob and obsk mice. n=6. (D) Total locomotive activity during the 48h metabolic cage monitor of 13 weeks old ob/ob and obsk mice. n=6. Data is represented as mean ± SD. Significance analysis was performed with unpaired Student’s *t*-test. *P < 0.05. ns, no significance.

**Figure S6. Sult2b1 ablation decreases serum fatty acid species and inhibits intestinal lipid uptake**

(A) Cluster heatmap of differential metabolites in serum of HFD-fed WT and Sult2b1KO mice. n=4. Arrow indicates the cluster of fatty acid (FA). **(B)** Relative mRNA expression of Sult2b1 in the liver, duodenum, jejunum, ileum, epididymal WAT, BAT, and soleus. n=3. Data is represented as mean ± SD. Significance analysis was performed with one-way ANOVA (multiple comparisons). **P < 0.01; ****P < 0.0001. **(C)** Eight weeks old male WT and Sult2b1KO mice were fed with control diet or HFD for 12 weeks. Sult2b1 protein levels in the duodenum and jejunum as measured by Western blotting. α-tubulin was used as a loading control. n=3. (D) Fluorescence visualization of lipid uptake in duodenum and jejunum of ob/ob and obsk mice 2h after an oral challenge of olive oil containing BODIPY-labeled FA. Scale bar, 100 µm. (E) Relative mRNA levels of fatty acid transporter genes *Cd36*, *Fatp4*, and *Fabppm* in the duodenum and jejunum in HFD-fed WT and Sult2b1KO mice. n=7. Data is represented as mean ± SD. Significance analysis was performed with unpaired Student’s *t*-test and multiple *t* tests. ns, no significance.

**Supplemental Table 1. Antibodies**

| **Antibodies/Reagents** | **Source** | **Identifier** |
| --- | --- | --- |
| Sult2b1 antibody | Santa Cruz | Cat# sc-166423  (1:600 dilution for WB) |
| Cd36 antibody | ABclonal | Cat# A1470  (1:1000 dilution for WB) |
| Phospho-Akt (Ser473) antibody | Cell Signaling Technology | Cat# 9271  (1:1000 dilution for WB) |
| Akt antibody | Cell Signaling Technology | Cat# 9272  (1:1000 dilution for WB) |
| F4/80 antibody | Cell Signaling Technology | Cat# 70076  (1:200 dilution for IHC) |
| Ucp1 antibody | Proteintech | Cat# 23673-1-AP  (1:1000/1:200 dilution for WB/IHC) |
| β-actin antibody | Sigma-Aldrich | Cat# A1978  (1:2000 dilution for WB) |
| α-tubulin antibody | Sigma-Aldrich | Cat# T6074  (1:2000 dilution for WB) |
| Anti-mouse IgG, HRP-linked | Cell Signaling Technology | Cat# 7076  (1:8000 dilution for WB) |
| Anti-rabbit IgG, HRP-linked | Cell Signaling Technology | Cat# 7074  (1:8000 dilution for WB) |

**Supplemental Table 2. Real-time PCR primer sequences.**

| **Mouse Gene Names** | **Sequences** |
| --- | --- |
| *Scd1* | Forward: 5’-TGCCCCTGCGGATCTT-3’ |
|  | Reverse: 5’-GCCCATTCGTACACGTCATT-3’ |
| *Acc2* | Forward: 5’-GAAGATGACAGACTCGAA-3’ |
|  | Reverse: 5’-CATCAGAGGAGTTGTCATC-3’ |
| *Cd36* | Forward: 5’-TCCTCTGACATTTGCAGGTCTATC-3’ |
|  | Reverse: 5’-AAAGGCATTGGCTGGAAGAA-3’ |
| *Cpt1a* | Forward: 5’-CTCAGTGGGAGCGACTCTTCA-3’ |
|  | Reverse: 5’-GGCCTCTGTGGTACACGACAA-3’ |
| *Sul2b1* | Forward: 5’-TGCTGGGCAATTAAAGGACC-3’ |
|  | Reverse: 5’-AGCCCTTGATGTGGTCAAAC-3’ |
| *Tnfa* | Forward: 5’-CATCTTCTCAAAATTCGAGTGACAA-3’ |
|  | Reverse: 5’-TGGGAGTAGACAAGGTACAACCC-3’ |
| *Il1b* | Forward: 5’-TTGAGGGACCCCAAAAGATG-3’ |
|  | Reverse: 5’-TGGACAGCCCAGGTCAAAG-3’ |
| *Il6* | Forward: 5’-TCCTCTCTGCAAGAGACTTCCATCC-3’ |
|  | Reverse: 5’-GGGAAGGCCGTGGTTGTCACC-3’ |
| *F4/80* | Forward: 5’- TTACGATGGAATTTCCTTGTATATCA-3’ |
|  | Reverse: 5’- CACAGCAGGAAGGTGGCTATG-3’ |
| *Cd68* | Forward: 5’-CCAATTCAGGGTGGAAGAAA-3’ |
|  | Reverse: 5’-CTCGGGCTCTGATGTAGGTC-3’ |

| **Mouse Gene Names** | **Sequences** |
| --- | --- |
| *Ucp1* | Forward: 5’-AAGCTGTGCGATGTCCATGT-3’ |
|  | Reverse: 5’-AAGCCACAAACCCTTTGAAAA-3’ |
| *Dio2* | Forward: 5’-CAGTGTGGTGCACGTCTCCAATC-3’ |
|  | Reverse: 5’-TGAACCAAAGTTGACCACCAG-3’ |
| *Ces1d* | Forward: 5’-GGAGAGTCAGCAGGAGGTTTC-3’ |
|  | Reverse: 5’-GAGGGACACACCACTCTCAG-3’ |
| *Ces1g* | Forward: 5’-TCGTGTCCCGTAGTCACAGA-3’ |
|  | Reverse: 5’-CAAAACTTGGGCGATACTGAT-3’ |
| *Abca1* | Forward: 5’-TCCTCATCCTCGTCATTCAAA-3’ |
|  | Reverse: 5’-GGACTTGGTAGGACGGAACCT-3’ |
| *Fatp4* | Forward: 5’-CCAGTAGTGTGGCCAACTTCCT-3’ |
|  | Reverse: 5’-CCACAGACCCACAAACTCATTG-3’ |
| *Fabppm* | Forward: 5’-AGCGGCTGACCAAGGAGTT-3’ |
|  | Reverse: 5’-GACCCCTGCCACGGAGAT-3’ |
